# Genome sequencing of the winged midge, *Parochlus steinenii*, from the Antarctic Peninsula

**DOI:** 10.1093/gigascience/giw009

**Published:** 2017-02-24

**Authors:** Sanghee Kim, Mijin Oh, Woongsic Jung, Joonho Park, Han-Gu Choi, Seung Chul Shin

**Affiliations:** 1 Division of Life Sciences, Korea Polar Research Institute (KOPRI), Incheon 21990, South Korea; 2 LabGenomics Clinical Research Institute, LabGenomics, Seongnam, Korea; 3 Department of Fine Chemistry, Seoul National University of Science and Technology, Seoul 01811, South Korea; 4 Unit of Polar Genomics, Korea Polar Research Institute (KOPRI), Incheon 21990, South Korea

**Keywords:** *Parochlus steinenii*, Cold-tolerant, Complete mitochondrial genome, Antarctic winged midge

## Abstract

**Background:** In the Antarctic, only two species of Chironomidae occur naturally—the wingless midge, *Belgica antarctica*, and the winged midge, *Parochlus steinenii*. *B. antarctica* is an extremophile with unusual adaptations. The larvae of *B. antarctica* are desiccation- and freeze-tolerant and the adults are wingless. Recently, the compact genome of *B. antarctica* was reported and it is the first Antarctic eukaryote to be sequenced. Although *P. steinenii* occurs naturally in the Antarctic with *B. antarctica*, the larvae of *P. steinenii* are cold-tolerant but not freeze-tolerant and the adults are winged. Differences in adaptations in the Antarctic midges are interesting in terms of evolutionary processes within an extreme environment. Herein, we provide the genome of another Antarctic midge to help elucidate the evolution of these species.

**Results:** The draft genome of *P. steinenii* had a total size of 138 Mbp, comprising 9513 contigs with an N50 contig size of 34,110 bp, and a GC content of 32.2%. Overall, 13,468 genes were predicted using the MAKER annotation pipeline, and gene ontology classified 10,801 (80.2%) predicted genes to a function. Compared with the assembled genome architecture of *B. antarctica*, that of *P. steinenii* was approximately 50 Mbp longer with 6.2-fold more repeat sequences, whereas gene regions were as similarly compact as in *B. antarctica*.

**Conclusions:** We present an annotated draft genome of the Antarctic midge, *P. steinenii*. The genomes of *P. steinenii* and *B. antarctica* will aid in the elucidation of evolution in harsh environments and provide new resources for functional genomic analyses of the order Diptera.

## Data description

### Sequencing

*Parochlus steinenii* specimens [1–3] were collected from King George Island, West Antarctica (62° 14^΄^ S, 58° 47^΄^ W) during 2014 and 2015. Twenty adults were used for genome sequencing, regardless of gender. Genomic DNA was extracted using a DNeasy Tissue Kit (Qiagen, Valencia, CA, USA). For genome sequencing and assembly using ALLPATHS-LG [4], two types of libraries were prepared. One was a fragment library, which was a paired-end type with an insert size of 400 bp (PE400), whereas others were jumping libraries, which were mate-pair types with insert sizes of 3 kbp (MP3K) and 5 kbp (MP5K). The paired-end library was sequenced with the MiSeq platform (Illumina, San Diego, CA, USA) using a read-length configuration of 2 × 300 bp, and the mate-pair libraries were sequenced with the HiSeq platform (Illumina, San Diego, CA, USA) using a read-length configuration of 2 × 150 bp (see Table 1). Library preparation and sequencing were performed according to the manufacturer's instructions.

**Table 1:** Sequencing libraries and respective yield used for genome assembly of *Parochlus steinenii*

		Insert	Library		Total read	
Library	Mode	size	type	Reads	lengths (Gbp)	Source
PE400	2 × 300	400	paired-end	51,892,430	15.6	Genomic DNA
MP3K	2 × 150	3000	mate-pair	170,887,140	25.6	Genomic DNA
MP5K	2 × 150	5000	mate-pair	157,622,418	23.6	Genomic DNA
PE300A	2 × 150	300	paired-end	27,663,170	3.5	RNA
PE300B	2 × 150	300	paired-end	27,782,288	3.5	RNA
PE300C	2 × 150	300	paired-end	30,806,804	3.9	RNA

For gene annotation with RNA evidence, total RNA was extracted from the whole bodies of ten adults in three different groups using the RNeasy Mini Kit (Qiagen, Valencia, CA, USA), according to the manufacturer's instructions. Three paired-end libraries with an insert size of 300 bp (PE300) were constructed using the TruSeq Stranded mRNA Library Prep Kit (Illumina, San Diego, CA, USA) and sequenced with the HiSeq platform (Illumina, San Diego, CA, USA) using a read-length configuration of 2 × 150 bp (Table 1).

Before assembly using ALLPATHS-LG, the paired-end reads resulting from the fragment library were trimmed using the fastq_quality_trimmer in the FASTX-Toolkit (Ver. 0.0.11) [5] with the parameters “-t 30”, “-l 200” and “-Q 33”. Paired sequences from the trimmed Illumina reads were then selected. Finally, after quality trimming, yields for the fragment library totaled 14.8 giga base pairs (Gbp).

Tree-type libraries were constructed in this study, as shown in Table 1. A PE400 library was constructed as a fragment library for ALLPATHS-LG. Mate-pair libraries (MP3K and MP5K) were also constructed for ALLPATHS-LG assembly. Three PE300 libraries (PE300A, PE300B, and PE300C) were constructed from RNA for gene annotation.

### Genome assembly

Before assembly, we estimated the genome size and heterozygosity using a k-mer analysis with sequencing reads. Jellyfish (Ver. 1.1.10) [6] and GenomeScope [7, 8] software were used. The 17-mers were counted in the reads from the PE400 library and the resulting histogram of 17-mer occurrence was used as a query for GenomeScope [8]. The estimated genome size was 143.8 mega base pairs (Mbp) and the estimated heterozygosity was 0.613%.

Assembly was performed using ALLPATHS-LG for both the fragment library (400 bp) and the jumping libraries (3 kbp and 5 kbp) [4]. This was performed on a 96-processor workstation with Intel Xeon X7460 2.66 GHz processors, 1 TB of RAM, and default parameters. For better assembly in ALLPATHS-LG, a larger k-mer size was used with one longer read generated from the paired-end library [4]. As a result, the paired-end reads from the fragment library were designed to overlap, and the insert size of the paired-end library was slightly less than twice the read size [4]. In this assembly, 93.8% of the paired-end reads from the fragment library overlapped and merged into one longer read. The resulting assembly had a total size of 138 Mbp, comprising 9513 contigs with an N50 contig size of 34,110 bp and an N50 scaffold size of 168 kbp (Table 2). The GC content was 32.2% and the assembly revealed contig coverage of approximately 89 × total read length from the fragment library. A total of 57.2% of the 3-kbp jumping library and 33.1% of the 5-kbp jumping library were used to improve scaffolding. If more jumping libraries or long jumping libraries (with insert size larger than 20 kbp) were used, the scaffolding might improve the assembly. The assembled genome size was similar to the predicted genome size (143.8 Mbp). We also validated this assembly using CEGMA [9] and BUSCO [10]. CEGMA evaluation showed that the gene completeness of this assembly was 85.08%, and BUSCO analysis using arthropod databases showed 67.2% completeness (Tables 3 and 4). If partially matched genes were considered, 92.34% and 87.5% of the genes were identified in CEGMA and BUSCO, respectively (Tables 3 and 4).

**Table 2:** Global statistics of the *Parochlus steinenii* genome assembly

Assembly results	Number	N50 (kbp)^a^	Size (Mbp)
Contig	9513	34.1	130.6
Scaffold	4151	168.1	138.0
Annotation	Number	Total length (kbp)	Percentage of the assembled genome
Genes	13,468	36,239.1	26.3
Coding regions (Coding regions in *B. antarctica*)	13,468 (11,005)	17,967.6 (17,518.0)	13.0 (19.6)
Introns (Introns in *B. antarctica*)	69,960 (43,577)	24,191.6 (15,494.9)	17.5 (17.2)
Repeats (Repeats in *B. antarctica*)	37,507 (10,084)	2252.6 (361.4)	1.6 (0.40)

a Minimum sequence length in which half of the assembled bases were found

**Table 3:** CEGMA analysis of two Antarctic midges

	CEG	Complete	Percentage	Total	Average	Percentage
	set	proteins	complete	observed	copy number	of orthologs
*P. steinenii*	Complete	211	85.08	247	1.17	14.22
	Partial	229	92.34	283	1.24	19.65
*B. antarctica*	Complete	241	97.18	283	1.17	12.03
	Partial	247	99.6	311	1.26	18.18

*CEG* core eukaryotic gene

**Table 4:** BUSCO analysis of two Antarctic midges

	Genome assembly		Gene set	
	*P. steinenii*	*B. antarctica*	*P. steinenii*	*B. antarctica*
Complete BUSCOs (%)	1798 (67.2)	2310 (86.4)	1890 (70.7)	2316 (86.6)
Complete and single-copy BUSCOs (%)	1648 (61.6)	2170 (81.1)	1620 (60.6)	2074 (77.5)
Complete and duplicated BUSCOs (%)	150 (5.6)	140 (5.2)	270 (10.1)	242 (9.0)
Fragmented BUSCOs (%)	543 (20.3)	270 (10.1)	343 (12.8)	137 (5.1)
Missing BUSCOs (%)	334 (12.5)	95 (0.04)	442 (16.5)	222 (8.3)
Total BUSCO groups searched	2675 (100)			

The statistics for gene annotation for *B. antarctica* were from a reanalysis for comparison of the percentage of the genome created, based on the assembled genome size. From a previous report [11], the assembled genome size of *B. antarctica* was 89.6 Mbp.

CEGMA analysis (Table 3) was performed to validate the genome assembly of *P. steinenii*. The genome sequence of *B. antarctica* (JPYR00000000.1) from the National Center for Biotechnology Information (NCBI) was also analyzed for comparison.

BUSCO analysis was performed to validate genome assembly and gene annotation. For *B. antarctica*, the genome sequence (JPYR00000000.1) from NCBI and the gene set annotated in this study were used. Table 4 shows the numbers and percentages of BUSCO groups.

### Repeat analysis and non-coding RNA

Interspersed repeats for *P. steinenii* were predicted using RepeatMasker (Ver. 3.3.0) [12] with a *de novo* repeat library. The *de novo* repeat library for *P. steinenii* was constructed using RepeatModeler (Ver. 1.0.3) [13], including the RECON (Ver. 1.07) [13] and RepeatScout (Ver. 1.0.5) [14] software, with default parameters. Tandem repeats, including simple repeats, satellites and low-complexity repeats, were predicted using TRF [15]. Putative tRNA genes were identified using tRNAscan-SE (Ver. 1.3.1) [16] with option “-H”. The repeat content for *B. antarctica* was re-estimated for comparison using RepeatMasker (Ver. 3.3.0) [12] with the Repbase library (Ver. 20140131) [17, 18]. The total coverage of repeat sequences in *P. steinenii* was approximately six times greater than that of repeat sequences in *B. antarctica* (Table 2), and the percentage of the genome was approximately three times higher than that of *B. antarctica*, based on the assembled genome size. Most statistics for repeats were higher in the *P. steinenii* library (Table 5). A total of 186 tRNAs were predicted through tRNAscan-SE [16] (Additional file 1: Table S1).

**Table 5:** Repeat content in Antarctic midges

	*P. steinenii*		*B. antarctica*	
	Total	Number	Total	Number
	coverage (bp)	of sequences	coverage (bp)	of sequences
Low complexity	404,490	8661	290,095	8812
Simple repeats	1,105,449	26,336	40,475	1066
				
**Transposon elements**				
Class I/LTR	289,059	1075	945	13
Class I/Non-LTR	169,298	675	18,003	271
Class II/DNA elements	216,807	649	5247	83
				
Small RNA	67,503	111	6425	13
Totals	2,252,606	37,507	361,370	10,258

*LTR* long terminal repeat

### Gene annotation

Gene annotation was accomplished using the MAKER annotation pipeline [19, 20]. RepeatMasker (Ver. 3.3.0) [12] was used to identify repetitive elements against a *de novo* repeat library, and the SNAP gene finder [21] was selected to perform *ab initio* gene prediction from the masked genome sequence. To find the best possible gene model for the given region, evidence of RNA and protein alignments were considered in MAKER2 [20]. Transcriptome assembly results were used for RNA evidence; the paired-end reads resulting from mRNA of the whole body of adults were trimmed using the fastq_quality_trimmer in the FASTX-Toolkit (Ver. 0.0.11) [5] with the parameters “-t 30”, “-l 80” and “-Q 33”, and they were assembled with CLC Genomics Workbench (Ver. 8.0.0) using default parameters. In all, 68,392 contigs, with an N50 contig size of 435 bp and an average contig size of 407 bp, were generated and used for RNA evidence. Protein sequences from six species, given in NCBI reference sequences, were used for protein evidence—*Drosophila melanogaster* (fruit fly, GCF_000001215.4), *Ceratitis capitata* (Mediterranean fruit fly, NC_000857.1), *Bactrocera dorsalis* (oriental fruit fly, NC_008748.1), *Anopheles gambiae* (African malaria mosquito, NZ_AAAB00000000.1), *Aedes aegypti* (yellow fever mosquito, AAGE00000000.2) and *Culex quinquefasciatus* (southern house mosquito, AAWU01000000). Alignment of transcriptome assembly with BLASTn and alignment of homologous protein information from tBLASTx were considered as evidence for annotation. To assess the annotated gene set, we ran a BUSCO analysis in the “OGS” mode for gene set completeness and identified 70.7% genes to be considered complete with the expanded gene set; 16.5% of the gene set was classified as missing [10].

Blast2GO (Ver. 2.6.0) assigned preliminary functions for 13,468 genes, and gene ontology (GO) classified 10,801 (80.2%) of the predicted genes to a function using the BLASTp and InterproScan results [22]. GO annotation described the classified proteins as those required for biological processes (7434; 55.2%), molecular functions (9576; 71.1%) and cellular components (4871; 36.2%). Enzyme Commission (EC) numbers were obtained for 987 proteins.

### Gene annotation for *B. antarctica*

To investigate the difference in gene content between *P. steinenii* and *B. antarctica*, we also annotated the genome of *B. antarctica* with the same methods used for *P. steinenii*. The reads in various experimental conditions for *B. antarctica* (SRR566981, SRR567289, SRR567164–SRR567167 and SRR567169–SRR567171) were downloaded from the NCBI Sequence Read Archive and we assembled them into 38,017 contigs, with an N50 contig size of 1799 bp and an average contig size of 913 bp, through CLC Genomics Workbench (Ver. 8.0.0). For RNA evidence, the resulting contigs were aligned to the genome sequence of *B. antarctica* with BLASTn in MAKER2. For protein evidence, we used the same protein sequence from the six species used for gene annotation in *P. steinenii* and predicted proteins of *B. antarctica*. From MAKER2, 11,005 genes were predicted in the *B. antarctica* genome. The annotated gene set in this analysis was assessed using BUSCO [10]. Gene set completeness was 86.6% including the expanded gene set, and 8.3% of the gene set was missing (Table 4).

### Ortholog analysis

**Figure 1:**
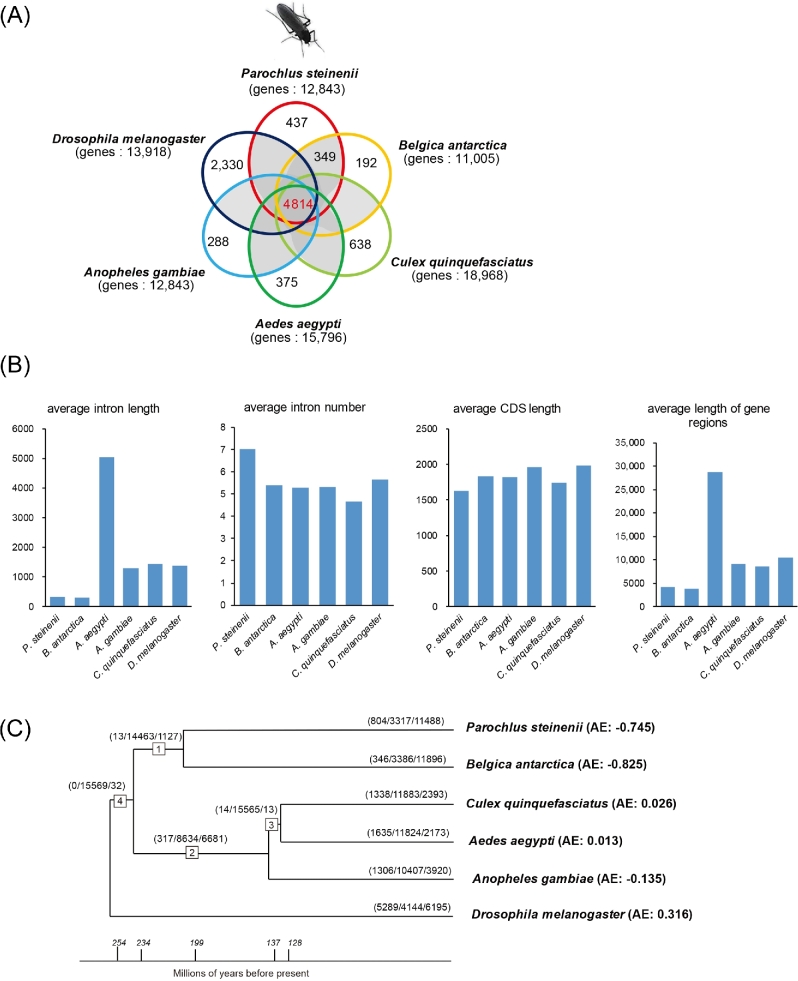
Genome-wide analysis of protein-coding genes in *Parochlus steinenii*. **a** Venn diagram displaying the overlap in orthologous genes of six insect species and the number of unique groups in each species. **b** The statistics of gene structures of the six insects. **c** Lineage-specific gene gains and losses among the six insects. The numbers in the *boxes* are identifiers for internal branches of the phylogeny. Numbers on each *branch* denote the number of gained, lost and stable genes, respectively. AE denotes the average expansion. The numbers on the *bottom line* denote the estimated divergence time of the corresponding tree nodes above, based on TimeTree

Orthologous groups were identified using OrthoMCL (Ver. 2.0.5) [23]. We used the standard parameters and options of OrthoMCL for all steps. In this analysis, coding sequences (CDS) from four genome assemblies (BDGP6 for *D. melanogaster*, AgamP4 for *A. gambiae*, AaegL3 for *A. aegypti* and CpipJ2 for *C. quinquefasciatus*) were collected from Ensemble Metazoa [24] and the CDS from MAKER2 were used for *B. antarctica* and *P. steinenii*. Total proteins were categorized into 15,633 groups—4814 orthologous groups were identified as common to all six insects, 437 groups specific to *P. steinenii* genes were not identified in any other species, and 349 groups were identified only in the two Antarctic midges (Fig. 1A and Additional file 1: Table S2).

### Gene structure of orthologous groups

*B. antarctica* showed a reduction in intron length with very low repeat sequences [11]. Therefore, we compared intron lengths of orthologous genes among the six insects to identify whether the intron lengths of the genes in *P. steinenii* were also reduced. We used the information from gene structures of the four genome assemblies (BDGP6 for *D. melanogaster*, AgamP4 for *A. gambiae*, AaegL3 for *A. aegypti* and CpipJ2 for *C. quinquefasciatus*) and the information from MAKER2 annotation of *B. antarctica* and *P. steinenii*. Among the six insects, the average intron length of *B. antarctica* (302 bp) was the smallest, although that of *P. steinenii* (319 bp) was similar (Fig. 1B). Despite a difference in the assembled genome size between *B. antarctica* and *P. steinenii* of approximately 50 Mbp, the average length of gene regions and CDS were also similar in the two. However, the average intron number in orthologous genes was higher in *P. steinenii*, which was the highest of all six insects (Fig. 1B).

### GO enrichment test

We used AgriGO [25] to identify which GO terms of the 437 groups that were unique to *P. steinenii* were statistically overrepresented relative to the GO terms of all genes of *P. steinenii*. A total of 1352 genes comprised these 437 groups, and therein were 717 genes with GO terms. AgriGO is a web-based tool for GO analysis: we selected “Fisher's exact test” for the statistical test method and “Hochberg FDR” as the multiple test adjustment method. GO terms were tested with a significance level of *p* < 0.05. Complete hierarchies of GO terms for each gene were examined. GO enrichment analysis identified 49 GO terms as statistically overrepresented: 26 GO terms in biological processes, five in cellular components and 18 in molecular functions (Table 6). It is noteworthy that 14 of the 26 significant GO terms in biological processes were associated with the unfolded protein response (UPR). The UPR is a stress response that occurs in the lumen of the endoplasmic reticulum (ER) [26]. When unfolded or misfolded proteins are accumulated in the ER lumen under stress conditions, the UPR is activated to improve protein folding by increasing the production of chaperones [26]. Representative GO terms in biological processes related to the UPR were mRNA splicing via endonucleolytic cleavage and ligation (GO:0070054), response to unfolded protein (GO:0006986), and endoplasmic reticulum unfolded protein response (GO:0030968).

**Table 6:** GO terms statistically overrepresented only in *Parochlus steinenii*-specific groups

GO	GO		No. of	No. of		
ID	tree	Term	genes^a^	genes^b^	*p*-value	FDR
GO:0006508	P	proteolysis	106	632	8.60E-13	2.60E-10
**GO:0006397**	P	mRNA processing	32	120	6.80E-10	1.00E-07
**GO:0070054**	P	mRNA splicing, via endonucleolytic cleavage and ligation	8	8	1.40E-09	1.40E-07
**GO:0016071**	P	mRNA metabolic process	32	130	5.80E-09	4.50E-07
**GO:0000394**	P	RNA splicing, via endonucleolytic cleavage and ligation	8	11	1.90E-07	1.10E-05
**GO:0006986**	P	response to unfolded protein	6	7	1.50E-06	7.60E-05
GO:0019538	P	protein metabolic process	173	1506	2.20E-06	9.40E-05
**GO:0051789**	P	response to protein stimulus	6	8	5.50E-06	0.00021
**GO:0006950**	P	response to stress	50	330	8.00E-06	0.00027
GO:0006468	P	protein amino acid phosphorylation	42	272	2.40E-05	0.00074
GO:0080135	P	regulation of cellular response to stress	9	24	4.80E-05	0.0013
**GO:0006396**	P	RNA processing	34	210	5.00E-05	0.0013
GO:0051347	P	positive regulation of transferase activity	8	22	0.00016	0.0031
GO:0033674	P	positive regulation of kinase activity	8	22	0.00016	0.0031
GO:0045860	P	positive regulation of protein kinase activity	8	22	0.00016	0.0031
**GO:0034620**	P	cellular response to unfolded protein	4	5	0.00017	0.0031
**GO:0030968**	P	endoplasmic reticulum unfolded protein response	4	5	0.00017	0.0031
GO:0042246	P	tissue regeneration	6	13	0.00024	0.0041
GO:0031099	P	regeneration	6	14	0.00039	0.0063
**GO:0071445**	P	cellular response to protein stimulus	4	6	0.00049	0.0071
**GO:0071216**	P	cellular response to biotic stimulus	4	6	0.00049	0.0071
**GO:0034976**	P	response to endoplasmic reticulum stress	4	7	0.0011	0.015
GO:0061053	P	somite development	3	4	0.0018	0.024
**GO:0006984**	P	ER-nuclear signaling pathway	4	8	0.002	0.026
GO:0006379	P	mRNA cleavage	4	9	0.0034	0.041
GO:0016310	P	phosphorylation	49	421	0.0041	0.049
						
GO:0031463	C	Cul3-RING ubiquitin ligase complex	5	5	2.90E-06	0.00019
GO:0031461	C	cullin-RING ubiquitin ligase complex	5	12	0.0014	0.047
GO:0005789	C	endoplasmic reticulum membrane	11	55	0.0032	0.063
GO:0042175	C	nuclear envelope–endoplasmic reticulum network	11	57	0.0042	0.063
GO:0044432	C	endoplasmic reticulum part	11	58	0.0049	0.063
						
GO:0004252	F	serine-type endopeptidase activity	76	292	3.70E-20	5.50E-18
GO:0004540	F	ribonuclease activity	30	54	1.90E-19	1.40E-17
GO:0008236	F	serine-type peptidase activity	76	318	6.90E-18	2.50E-16
GO:0017171	F	serine hydrolase activity	76	318	6.90E-18	2.50E-16
GO:0004175	F	endopeptidase activity	84	416	5.70E-15	1.70E-13
GO:0070011	F	peptidase activity, acting on L-amino acid peptides	103	570	1.60E-14	4.00E-13
GO:0008233	F	peptidase activity	103	595	2.40E-13	5.10E-12
GO:0004518	F	nuclease activity	30	102	1.70E-10	3.10E-09
GO:0031072	F	heat shock protein binding	10	17	1.00E-07	1.60E-06
GO:0004672	F	protein kinase activity	47	300	5.90E-06	8.70E-05
GO:0008234	F	cysteine-type peptidase activity	15	59	3.70E-05	0.00049
GO:0016787	F	hydrolase activity	171	1580	5.00E-05	0.00061
GO:0016773	F	phosphotransferase activity, alcohol group as acceptor	49	363	0.00018	0.002
GO:0042802	F	identical protein binding	10	38	0.00052	0.0055
GO:0031625	F	ubiquitin protein ligase binding	5	12	0.0014	0.014
GO:0005515	F	protein binding	229	2357	0.0015	0.014
GO:0016301	F	kinase activity	48	405	0.0032	0.027
GO:0003676	F	nucleic acid binding	144	1469	0.0055	0.045

A total of 49 GO terms were statistically overrepresented: 26 in biological processes (P), five in cellular components (C) and 18 in molecular functions (F) were identified as significant by GO enrichment analysis. Fisher's exact test was performed and the resulting *p*-values were adjusted using the Hochberg FDR for multiple comparisons. GO terms associated with the unfolded protein response are shown in *bold font*

*ER* endoplasmic reticulum, *FDR* false discovery rate, *GO* gene ontology

a The number of genes with GO terms in the *P. steinenii*-specific groups

b The number of genes with GO terms in *P. steinenii*'s entire gene set

### Likelihood analysis of gene gain and loss

To estimate the average gene expansion/contraction rate and to identify gene families that have undergone significant size changes through evolution [27, 28], we estimated differences in the size of 15,633 orthologs using the program CAFE3.0 [29]. A Newick description of a rooted and bifurcating phylogenetic tree was needed for this analysis. Therefore, we performed phylogenetic analyses among six insects with the protein-coding gene in the orthologous groups. We selected 4814 orthologous gene sets from the orthologous groups from OrthoMCL using the criterion of reciprocal best BLASTP hit. Protein-coding gene sequences were aligned using PRANK (Ver. 130820) under a codon model with the “-dna -codon” option [30], poor alignment sites were eliminated using Gblock (Ver. 0.91) under a codon model with the “-t = c” option [31], and the remaining alignment regions were concatenated for use in the phylogenetic analyses. The phylogenetic tree was constructed using the neighbor-joining method [32] in the MEGA (Ver. 6) program [33]. From the resulting phylogenetic tree, we prepared the ultrametric tree of the species, including branch lengths in units of time through TimeTree [34], for the analysis (Fig. 1C). We ran the program using *p* < 0.05, and estimated rates of birth (λ) and death (μ) were calculated using the program LambdaMu with the “-s” option. We calculated the number of gene gains and losses on each branch of the tree with the “-t” option. The average expansion (AE) sizes of the two Antarctic midges were lower than those of the other four insects (Fig. 1C), and *D. melanogaster* exhibited the highest AE score among the six. Using *p* < 0.0001 for the family-wide significance value, we expected approximately one significant result by chance and calculated the exact *p*-values for transitions over every branch. We called individual branches significant at *p* < 0.005 [35]. We identified three gene families that were significantly expanded in *P. steinenii* and two in *B. antarctica*, (Additional file 1: Table S3).

## Availability of supporting data

Supporting data (sequence files for CDS, proteins, transcripts and the draft genome, and the general feature format for genes and repeats) are available in the *GigaScience* GigaDB database [36] and the raw data were deposited in the NCBI BioProject repository PRJNA284858 (SRX1976250–SRX1976255).

## Additional file

**Additional file 1: Table S1** tRNA in *Parochlus steinenii*. (DOCX 21 kb)

**Additional file 1: Table S2** Shared orthologous gene clusters among six insects—*Drosophila melanogaster, Anopheles gambiae, Aedes aegypti, Culex quinquefasciatus, Belgica antarctica* and *Parochlus steinenii*

**Additional file 1: Table S3** Gene families were significantly expanded in Antarctic midges

## Abbreviations

CDS, coding sequence; Gbp, giga base pairs; GO, gene ontology; Mbp, mega base pairs.

## Competing interests

The authors declare that they have no competing interests.

## Authors' contributions

SK, HGC and SCS designed the study. SK, WJ and HGC collected the samples and performed the experiments. SCS and JP analyzed the data. All authors participated in the writing of the manuscript.

## Supplementary Material

Additional files **Additional file 1: Table S1** tRNA in *Parochlus steinenii*. (DOCX 21 kb)
**Additional file 1: Table S2** Shared orthologous gene clusters among six insects—*Drosophila melanogaster, Anopheles gambiae, Aedes aegypti, Culex quinquefasciatus, Belgica antarctica* and *Parochlus steinenii*
**Additional file 1: Table S3** Gene families were significantly expanded in Antarctic midges

GIGA-D-16-00062_Original_Submission.pdf

GIGA-D-16-00062_Revision_1.pdf

Response_to_Reviewers_Original_Submission.pdf

Reviewer_1_Report_(Original_Submission).pdf

Reviewer_1_Report_(Revision_1).pdf

Reviewer_2_Report_(Original_Submission).pdf

Reviewer_3_Report_(Original_Submission).pdf

Reviewer_3_Report_(Revision_1).pdf

Reviewer_4_Report_(Original_Submission).pdf
