# Supplementary material for: Genome sequencing of the winged midge, Parochlus steinenii, from the Antarctic Peninsula
Source: Gigascience. 2017 Feb 24;6(3):1–8. doi: 10.1093/gigascience/giw009 (PMC5467013; doi:10.1093/gigascience/giw009)
Supplement: Additional files — Additional file 1: Table S1 tRNA in Parochlus steinenii. (DOCX 21 kb) Additional file 1: Table S2 Shared orthologous gene clusters among six insects—Drosophila melanogaster, Anopheles gambiae, Aedes aegypti, Culex quinquefasciatus, Belgica antarctica and Parochlus steinenii Additional file 1: Table S3 Gene families were significantly expanded in Antarctic midges [file giw009_Supp.docx]

**Table S1 tRNA in *Parochlus steinenii***

| **Anticodon** | **Number** |
| --- | --- |
| Ala | 4 |
| Arg | 13 |
| Asn | 5 |
| Asp | 5 |
| Cys | 3 |
| Gln | 9 |
| Glu | 15 |
| Gly | 9 |
| His | 9 |
| Ile | 8 |
| Leu | 13 |
| Lys | 7 |
| Met | 7 |
| Phe | 5 |
| Pro | 7 |
| Pseudo | 15 |
| SeC(e) | 1 |
| Ser | 13 |
| Thr | 13 |
| Trp | 3 |
| Tyr | 9 |
| Val | 13 |
| **Total** | **186** |

186 tRNAs were predicted using tRNAscan-SE

**Table S2 Shared orthologous gene clusters among six insects—*Drosophila melanogaster, Anopheles gambiae, Aedes aegypti, Culex quinquefasciatus, Belgica antarctica* and *Parochlus steinenii***

| **Group** | **Number** |  | **Group** | **Number** |  | **Group** | **Number** |
| --- | --- | --- | --- | --- | --- | --- | --- |
| A | 437 |  | ACDF | 32 |  | BDE | 6 |
| AB | 349 |  | ACE | 15 |  | BDEF | 31 |
| ABC | 18 |  | ACEF | 23 |  | BDF | 2 |
| ABCD | 46 |  | ACF | 4 |  | BE | 6 |
| ABCDE | 452 |  | AD | 18 |  | BEF | 6 |
| ABCDEF | 4814 |  | ADE | 12 |  | BF | 33 |
| ABCDF | 84 |  | ADEF | 22 |  | C | 638 |
| ABCE | 24 |  | ADF | 3 |  | CD | 1196 |
| ABCEF | 102 |  | AE | 15 |  | CDE | 1258 |
| ABCF | 8 |  | AEF | 9 |  | CDEF | 359 |
| ABD | 9 |  | AF | 46 |  | CDF | 50 |
| ABDE | 20 |  | B | 192 |  | CE | 105 |
| ABDEF | 190 |  | BC | 28 |  | CEF | 20 |
| ABDF | 8 |  | BCD | 34 |  | CF | 31 |
| ABE | 11 |  | BCDE | 130 |  | D | 375 |
| ABEF | 37 |  | BCDEF | 682 |  | DE | 114 |
| ABF | 69 |  | BCDF | 22 |  | DEF | 17 |
| AC | 71 |  | BCE | 9 |  | DF | 25 |
| ACD | 65 |  | BCEF | 25 |  | E | 288 |
| ACDE | 158 |  | BCF | 5 |  | EF | 25 |
| ACDEF | 410 |  | BD | 10 |  | F | 2330 |
|  |  |  |  |  |  | **Total** | **15,633** |

Shared orthologous gene clusters among six insects were calculated using OrthoMCL with default parameters. Coding sequences from *D. melanogaster* (13,918), *A. gambiae* (12,843), *A. aegypti* (15,796), *C. quinquefasciatus* (18,968), *B. antarctica* (11,005) and *P. steinenii* (12,843) were used for ortholog analysis

*A Parochlus steinenii*, *B Belgica antarctica*, *C Culex quinquefasciatus*, *D Aedes aegypti*, *E Anopheles gambiae*, *F* *Drosophila melanogaster*

**Table S3 Gene families were significantly expanded in Antarctic midges**

| **ID** | **Annotation** | **Size** | | | | | | | | | | **Family-wide *p*-values** | **Viterbi *p*-values** | | | | | | | | | |
| --- | --- | --- | --- | --- | --- | --- | --- | --- | --- | --- | --- | --- | --- | --- | --- | --- | --- | --- | --- | --- | --- | --- |
|  |  | ***P. steinenii*** | ***B. antarctica*** | **1*** | **2*** | ***C. quinquefasciatus*** | ***A. aegypti*** | **3*** | ***A. gambiae*** | **4*** | ***D. melanogaster*** |  | ***P. steinenii*** | ***B. antarctica*** | **1*** | **2*** | ***C. quinquefasciatus*** | ***A. aegypti*** | **3*** | ***A. gambiae*** | **4*** | ***D. melanogaster*** |
| PS0025 | Leucine-rich membrane protein | 42 | 0 | 0 | 0 | 0 | 0 | 0 | 0 | 1 | 0 | 0 | 0 | 0.625 | 0.073 | 0.161 | 0.5 | 0.5 | 0.5 | 0.5 | 0.509 | 0.14 |
| PS0032 | Clip-domain serine protease | 40 | 0 | 0 | 0 | 0 | 0 | 0 | 0 | 1 | 0 | 0 | 0 | 0.625 | 0.073 | 0.161 | 0.5 | 0.5 | 0.5 | 0.5 | 0.509 | 0.14 |
| PS0098 | Zinc finger protein | 26 | 1 | 0 | 0 | 0 | 0 | 0 | 0 | 1 | 0 | 0 | 0 | 0.875 | 0.073 | 0.161 | 0.5 | 0.5 | 0.5 | 0.5 | 0.509 | 0.14 |
| PS0074 | Serine protease gd-like | 0 | 29 | 0 | 0 | 0 | 0 | 1 | 0 | 1 | 0 | 0 | 0.625 | 0 | 0.073 | 0.161 | 0.5 | 0.5 | 0.5 | 0.5 | 0.509 | 0.14 |
| PS0114 | Leucine-rich repeat-containing protein | 0 | 26 | 0 | 0 | 0 | 0 | 1 | 0 | 1 | 0 | 0 | 0.625 | 0 | 0.073 | 0.161 | 0.5 | 0.5 | 0.5 | 0.5 | 0.509 | 0.14 |

*Identifiers for internal branches of the phylogeny (see Figure 1C)
