# Supplementary material for: Genome sequencing of the winged midge, Parochlus steinenii, from the Antarctic Peninsula
Source: Gigascience. 2017 Feb 24;6(3):1–8. doi: 10.1093/gigascience/giw009 (PMC5467013; doi:10.1093/gigascience/giw009)
Supplement: GIGA-D-16-00062_Original_Submission.pdf [file giw009_GIGA-D-16-00062_Original_Submission.pdf]

# GigaScience

## Genome sequencing of the winged midge, Parochlus steinenii, from the Antarctic Peninsula

--Manuscript Draft--

| | |
|---|---|
| **Manuscript Number:** | GIGA-D-16-00062 |
| **Full Title:** | Genome sequencing of the winged midge, Parochlus steinenii, from the Antarctic Peninsula |
| **Article Type:** | Data Note |
| **Funding Information:** | Korea Polar Research Institute (PE16070) — Dr Hyun Park<br>Korea Polar Research Institute (PE16080) — Not applicable |
| **Abstract:** | Background<br>In the Antarctic, only two species of Chironomidae occur naturally: the wingless midge, Belgica antarctica, and the winged midge, Parochlus steinenii. B. antarctica has unusual characteristics and it has adapted to an extreme environment. The larvae of B. antarctica are desiccation and freeze tolerant, and the adults lose their wings. Recently, a study suggested that the compact genome of B. antarctica could be the result of adaptation to an extreme environment. On the other hand, P. steinenii, is cold tolerant but not freeze tolerant at the larval stage, even though it occurs naturally in the Antarctic with B. antarctica. In addition, P. steinenii adults are winged. As a result, P. steinenii could be a good species for comparative analysis in order to understand the notable adaptations of B. antarctica. In this study, we sequenced the genome of P. steinenii.<br>Results<br>The draft genome of P. steinenii had a total size of 137 Mb, comprising 9,513 contigs with an N50 contig size of 34,110 bp, and a GC content of 32.2%. The assembled contig had a contig coverage of approximately 108.5×. In all, 13,468 genes were predicted using MAKER annotation pipeline and classified to functions for 10,801 (80.2%) predicted genes in gene ontology.<br>Conclusions<br>We present an annotated draft genome of the Antarctic midge, P. steinenii. The P. steinenii genome will help reveal the mechanism underlying freeze tolerance when compared with the genome of B. antarctica, as P. steinenii is cold tolerant but not freeze tolerant in the larval form. |
| **Corresponding Author:** | Seung Chul Shin, Ph.D<br><br>KOREA, REPUBLIC OF |
| **Corresponding Author Secondary Information:** | |
| **Corresponding Author's Institution:** | |
| **Corresponding Author's Secondary Institution:** | |
| **First Author:** | Sanghee Kim |
| **First Author Secondary Information:** | |
| **Order of Authors:** | Sanghee Kim<br>Mijin Oh<br>Woongsic Jung<br>Joonho Park<br>Han-Gu Choi<br>Hyun Park |

Powered by Editorial Manager® and ProduXion Manager® from Aries Systems Corporation

| | |
|---|---|
| | Seung Chul Shin, Ph.D |
| **Order of Authors Secondary Information:** | |
| **Opposed Reviewers:** | |
| **Additional Information:** | |
| **Question** | **Response** |
| Are you submitting this manuscript to a special series or article collection? | No |
| **Experimental design and statistics**<br><br>Full details of the experimental design and statistical methods used should be given in the Methods section, as detailed in our Minimum Standards Reporting Checklist. Information essential to interpreting the data presented should be made available in the figure legends.<br><br>Have you included all the information requested in your manuscript? | Yes |
| **Resources**<br><br>A description of all resources used, including antibodies, cell lines, animals and software tools, with enough information to allow them to be uniquely identified, should be included in the Methods section. Authors are strongly encouraged to cite Research Resource Identifiers (RRIDs) for antibodies, model organisms and tools, where possible.<br><br>Have you included the information requested as detailed in our Minimum Standards Reporting Checklist? | Yes |
| **Availability of data and materials**<br><br>All datasets and code on which the conclusions of the paper rely must be either included in your submission or deposited in publicly available repositories (where available and ethically appropriate), referencing such data using a unique identifier in the references and in the "Availability of Data and Materials" section of your manuscript.<br><br>Have you have met the above requirement as detailed in our Minimum Standards Reporting Checklist? | Yes |

Powered by Editorial Manager® and ProduXion Manager® from Aries Systems Corporation

Manuscript

Click here to view linked References

Click here to download Manuscript Parochlus_manuscript_2nd.docx

# Genome sequencing of the winged midge, *Parochlus steinenii*, from the Antarctic Peninsula

Sanghee Kim[1], Mijin Oh[4], Woongsic Jung[1], Joonho Park[3], Han-Gu Choi[1], Hyun Park[1,2], and Seung Chul Shin[1*]

[1]Division of Life Sciences, Korea Polar Research Institute (KOPRI), [2]Department of Polar Sciences, Korea University of Science and Technology, Incheon 21990, Republic of Korea, [3]Department of Fine Chemistry, Seoul National University of Science and Technology, Seoul, South Korea,[4]LabGenomics Clinical Research Institute, LabGenomics, Seongnam, Korea.

Keywords: *Parochlus steinenii*, complete mitochondrial genome, Antarctic winged midge

* Corresponding author

Name: SEUNG CHUL SHIN

Address: Division of Polar Life Sciences, Korea Polar Research Institute, 26 Songdomirae-ro, Yeonsu-gu, Incheon 21990, Republic of Korea

Phone: +82-32-760-5572

Fax: +82-32-760-5598

E-mail: ssc@kopri.re.kr

## Abstract

### Background

In the Antarctic, only two species of Chironomidae occur naturally—the wingless midge, *Belgica antarctica*; and the winged midge, *Parochlus steinenii*. *B. antarctica* has unusual characteristics with a compact genome as a result of adaptation to an extreme environment. The larvae of *B. antarctica* are desiccation and freeze tolerant and the adults lose their wings. Even though they occur naturally in the Antarctic with *B. antarctica*, the larvae of *P. steinenii* are cold, but not freeze, tolerant and the adults are winged. Therefore, *P. steinenii* could be a good species for comparative analysis in order to understand the notable adaptations of *B. antarctica*. In this study, we sequenced the genome of *P. steinenii*.

### Results

The draft genome of *P. steinenii* had a total size of 137 Mbp, comprising 9,513 contigs with an N50 contig size of 34,110 bp and a GC content of 32.2%. In all, 13,468 genes were predicted using MAKER annotation pipeline, and gene ontology classified 10,801 (80.2%) predicted genes to a function. As compared to genome architecture of *B. antarctica*, that of *P. steinenii* was 39 Mbp longer with 4-fold increased repeat sequences, whereas gene regions were similarly compact as *B. antarctica*.

### Conclusions

We present an annotated draft genome of the Antarctic midge, *P. steinenii*. The *P. steinenii* genome will help reveal the mechanism underlying freeze tolerance when compared to the genome of *B. antarctica*, as *P. steinenii* is cold, but not freeze, tolerant in the larval form.



### Keywords

*Parochlus steinenii*, cold tolerant, Antarctic midge

## Data description

### Sequencing

Specimen of *Parochlus steinenii* [1-3] was collected from King George Island, West Antarctica (62°14′S, 58°47′W) during 2014 and 2015. Genomic DNA was extracted using a DNeasy Tissue Kit (Qiagen, Valencia, CA, USA). For genome sequencing and assembly using ALLPATHS-LG [4], two types of libraries were prepared. One was a fragment library, which was of paired-end type with an insert size of 400 bp, while the other was a jumping library, which was of mate-pair type with insert sizes of 3 kbp and 5 kbp. Paired-end libraries were sequenced with the MiSeq platform (Illumina, San Diego, CA, USA) using a read length configuration of 2 × 300 bp, and mate-pair libraries were sequenced with the Illumina HiSeq platform (Illumina, San Diego, CA, USA) using a read length configuration of 2 × 150 bp (see Table 1). Library preparation and sequencing were performed according to the manufacturer's instructions.

For gene annotation with expressed sequencing tags, RNA was extracted from whole body of *P. steinenii* using the Qiagen kit, according to the manufacturer's instructions. Paired-end libraries with the insert size of 300 bp were constructed and sequenced with the Illumina HiSeq platform (Illumina, San Diego, CA, USA), using a read length configuration of 2 × 150 bp (Table 1).

Before assembly using ALLPATH-LG, the paired-end reads resulting from the fragment library were trimmed using the FASTX-Toolkit (Ver. 0.0.11) (http://hannonlab.cshl.edu/fastx_toolkit) with the parameters -t 30, -l 200, and -Q 33. Paired sequences from the trimmed Illumina reads were then selected. Finally, data from paired-end trimmed reads with 14 gigabase pairs (Gbp) were obtained (Table 1).

**Table 1. Sequencing statistics of *P. steinenii***

| Library | Mode | Insert size | Library type | Reads | Read lengths | Source |
|---|---|---|---|---|---|---|
| PE400trim | 2×300 | 400 | paired-end | 51 648 324 | 14 775 480 106 | Genomic DNA |
| PE400 | 2×300 | 400 | paired-end | 51 892 430 | 15 567 729 000 | Genomic DNA |
| MP3K | 2×150 | 3 000 | mate-pair | 170 887 140 | 25 633 071 000 | Genomic DNA |
| MP5K | 2×150 | 5 000 | mate-pair | 157 622 418 | 23 643 362 700 | Genomic DNA |
| PE300 | 2×150 | 300 | paired-end | 27 663 170 | 3 539 060 573 | RNA |
| PE300 | 2×150 | 300 | paired-end | 27 782 288 | 3 483 157 066 | RNA |
| PE300 | 2×150 | 300 | paired-end | 30 806 804 | 3 875 228 963 | RNA |

## Genome assembly

Assembly was performed using ALLPATHS-LG for both, the fragment libraries (400 bp) and the jumping libraries (3 kbp and 5 kbp). These were performed on a 96-processor workstation with Intel Xeon X7460 2.66 GHz processors, 1 terabyte RAM, and default parameters. In ALLPATHS-LG, paired-end reads from the fragment library were merged to make longer reads, resulting in a better assembly and a larger k-mer size [4]. As a result, the fragment library should be designed to overlap, and the size of the paired-end library was slightly less than twice the read size [4]. In this assembly, 93.8% of the fragment library was full. The resulting assembly had a total size of 137 Mb, comprising 9,513 contigs, with an N50 contig size of 34,110 bp, and an N50 scaffold size of 168 kb (Table 2). The GC content was 32.2% and the assembly revealed contig coverage of approximately 108.5 ×.

**Table 2. Global statistics of the *P. steinenii* genome assembly.**

| Assembly results | Number | N50 (kb) * | Size (Mb) |
|---|---|---|---|
| Contig | 9 513 | 34.1 | 130.6 |
| Scaffold | 4 151 | 168.1 | 138.0 |

| Annotation | Number | Total length (kb) | Percentage of genome (%) |
|---|---|---|---|
| Genes | 13 468 | 36 239.1 | 26.3 |
| Coding region (Coding regions in *B. antarctica*) | 13 468 (13 517) | 17 967.6 (18 964.3) | 13.0 (19.4) |
| Introns (Introns in *B. antarctica*) | 69 960 (43 577) | 24 191.6 (15 495.0) | 17.5 (15.7) |
| Repeats (Repeats in *B. antarctica*) | 37 507 (10 084) | 2 252.6 (429.7) | 1.6 (0.49) |

*Minimum sequence length in which half of the assembled bases were found. The statistics of gene annotation of *B. antarctica* are quoted from a previously reported paper [5].

**Gene annotation**

Gene annotation was accomplished using MAKER2 annotation pipeline [6]. RepeatMasker (Ver. 3.3.0) [7] was used to identify repetitive elements against a *de novo* repeat library, and the SNAP gene finder [8] was selected to perform *ab initio* gene prediction from the masked genome sequence in MAKER2. For proper gene annotation, RNA and protein evidence alignment were used. Alignment of expressed sequence tags with BLASTn and homologous protein information from tBLASTx were considered for evidence of alignment. Transcriptome assembly results were used for RNA evidence, and a CLC Genomics Workbench (Ver. 8.0.0) was used for assembly. In all, 68,392 contigs with an N50 contig size of 435 bp and an average contig size of 407 bp, were generated.

Protein sequences from six species, given in NCBI reference sequences, were used in the analysis—*Drosophila melanogaster* (Fruit fly, GCF_000001215.4), *Ceratitis capitata* (Mediterranean fruit fly, NC_000857.1), *Bactrocera dorsalis* (oriental fruit fly, NC_008748.1), *Anopheles gambiae* (African malaria mosquito, NZ_AAAB00000000.1), *Aedes aegypti* (yellow fever mosquito, AAGE00000000.2), and *Culex quinquefasciatus* (southern house mosquito, AAWU01000000). A total of 13,468 genes in the *P. steinenii* genome were predicted using the MAKER2 pipeline. This is similar to the number of genes in *B. antarctica* [5]. The compact genome of *B. antarctica* (99 Mbp) [5], which is endemic to Antarctica, notably comprises of low repeat density and a reduced intron length. Although *P. steinenii* showed a low repeat density (1.6%; Table 2), it was not as low as that of *B. antarctica*, but it does have a similar intron length in a percentage of genome [5].

Blast2Go (Ver. 2.6.0) assigned preliminary functions for 13,468 genes, and gene ontology (GO) classified 10,801 (80.2%) of the predicted genes to a function. This was annotated with the BLASTp results and InterproScan [9]. GO annotation described the classified proteins as those required for biological processes (7,434, 55.2%) and molecular functions (9,576, 71.1%), and as cellular components (4,871, 36.2%). Enzyme commission (EC) numbers were obtained for 987 proteins.

**Gene annotation for *B. antarctica***

To investigate the difference in gene contents between *P. steinenii* and *B. antarctica*, we also annotated the genome of *B. antarctica* with the same methods used for *P. steinenii*. For RNA evidence alignment in MAKER2 annotation pipe lines [6], the reads in various experimental conditions with *B. antarctica* (SRR566981, SRR567289, SRR567164~7, SRR567169~71) were downloaded from SRA databases in NCBI and we assembled the reads into 38,017

contigs with an N50 contig size of 1,799 bp and an average contig size of 913 bp through CLC Genomics Workbench (Ver. 8.0.0).

We matched proteins from *P. steinenii* to those from six other species for protein evidence. From MAKER2, a total of 11,005 genes were predicted in the *B. antarctica* genome and were used for ortholog analysis.

**Repeat analysis and Non-coding RNA**

Interspersed repeats were predicted using RepeatMasker (Ver. 3.3.0) with a *de novo* repeat library [7]. A *de novo* repeat library was constructed using RepeatModeler (Ver. 1.0.3) [10], including the RECON (Ver. 1.07) [10] and RepeatScout (Ver. 1.0.5) [11] software, with default parameters, and tandem repeats including simple repeats, satellites, and low complexity repeats were predicted using TRF [12]. Putative tRNA genes were identified using tRNAscan-SE (Ver. 1.3.1) [13] with option -H. The total coverage of repeat sequences in *P. steinenii* were up to approximately four-fold from those of repeat sequences in *B. antarctica* (Table 2), and the percentage of genome was increased approximately three-fold as compared to that of *B. antarctica*. Most statistics of repeats were increased in the library of *P. steinenii* (Table 3). Through tRNAscan-SE, 186 tRNAs were predicted (Table 4).

**Table 3. Repeat content in Antarctic midges**

| | ***P. steinenii*** | | ***B. antarctica*** | |
|---|---|---|---|---|
| | **Total coverage (bp)** | **Number of sequences** | **Total coverage (bp)** | **Number of sequences** |
| Low complexity | 404 490 | 8 661 | 276 261 | 8 536 |

| | | | | |
|---|---|---|---|---|
| Simple repeats | 1 105 449 | 26 336 | 36 911 | 999 |
| | | | | |
| Transposon elements | | | | |
| Class I/LTR | 289 059 | 1 075 | 74 297 | 336 |
| Class I/Non-LTR | 169 298 | 675 | 26 554 | 128 |
| Class II/DNA elements | 216 807 | 649 | 8 536 | 64 |
| | | | | |
| Small RNA | 67 503 | 111 | 7 165 | 36 |
| Total | 2 252 606 | 37 507 | 429 724 | 10 069 |

The statistics of repeats of *B. antarctica* are quoted from a previously reported paper [5].

**Table 4. tRNA in *P. steinenii***

| **Anticodon** | **number** |
|---|---|
| Ala | 4 |
| Arg | 13 |
| Asn | 5 |
| Asp | 5 |
| Cys | 3 |
| Gln | 9 |
| Glu | 15 |
| Gly | 9 |
| His | 9 |
| Ile | 8 |

| | |
|---|---|
| Leu | 13 |
| Lys | 7 |
| Met | 7 |
| Phe | 5 |
| Pro | 7 |
| Pseudo | 15 |
| SeC(e) | 1 |
| Ser | 13 |
| Thr | 13 |
| Trp | 3 |
| Tyr | 9 |
| Val | 13 |
| **sum** | **186** |

**Ortholog analysis.**

Orthologous groups were identified using OrthoMCL (Ver. 2.0.5) [14]. We used the standard parameters and options of OrthoMCL for all steps. In this analysis, coding sequences (CDS) from six insects (*D. melanogaster*, *A. gambiae*, *A. aegypti, C. quinquefasciatus, B. antarctica, and P. steinenii*) were used. In this study, CDS from four genome assemblies (BDGP6 for *D. melanogaster*, AgamP4 for *A. gambiae*, AaegL3 for *A. aegypti*, and CpipJ2 for *C. quinquefasciatus*) were collected from Ensemble Metazoa (http://metazoa.ensembl.org/index.html) and the CDS from MAKER2 were used for *B. antarctica and P. steinenii*. Total proteins were categorized into 15,633 groups—4,814 orthologous groups were identified as common to all the six insects, 437 orthologous groups in *P. steinenii* genes were not identified in any other species, and 349 groups were identified only in the two Antarctic midges (Fig. 1A and Table 5).

**Table 5. Shared orthologous gene clusters among six insects—*D. melanogaster, A. gambiae, A. aegypti, C. quinquefasciatus, B. antarctica,* and *P. steinenii.***

| Group | Number | Group | Number |
|---|---|---|---|
| A | 437 | B | 192 |
| AB | 349 | BC | 28 |
| ABC | 18 | BCD | 34 |
| ABCD | 46 | BCDE | 130 |
| ABCDE | 452 | BCDEF | 682 |
| ABCDEF | 4 814 | BCDF | 22 |
| ABCDF | 84 | BCE | 9 |
| ABCE | 24 | BCEF | 25 |

| | | | |
|---|---|---|---|
| ABCEF | 102 | BCF | 5 |
| ABCF | 8 | BD | 10 |
| ABD | 9 | BDE | 6 |
| ABDE | 20 | BDEF | 31 |
| ABDEF | 190 | BDF | 2 |
| ABDF | 8 | BE | 6 |
| ABE | 11 | BEF | 6 |
| ABEF | 37 | BF | 33 |
| ABF | 69 | C | 638 |
| AC | 71 | CD | 1 196 |
| ACD | 65 | CDE | 1 258 |
| ACDE | 158 | CDEF | 359 |
| ACDEF | 410 | CDF | 50 |
| ACDF | 32 | CE | 105 |
| ACE | 15 | CEF | 20 |
| ACEF | 23 | CF | 31 |
| ACF | 4 | D | 375 |
| AD | 18 | DE | 114 |
| ADE | 12 | DEF | 17 |
| ADEF | 22 | DF | 25 |
| ADF | 3 | E | 288 |
| AE | 15 | EF | 25 |
| AEF | 9 | F | 2 330 |
| AF | 46 | **Total** | **15 633** |

A: *P. steinenii*, B: *B. antarctica*, C: *C. quinquefasciatus*, D: *A. aegypti*, E: *A. gambiae*, and F: *D. melanogaster*

## Gene structure of Orthologous groups

*B. antarctica* showed a reduction in intron length with very low repeat sequences [5], so we compared intron lengths of orthologous gene among six insects to identify whether the intron length of the gene in *P. steinenii* was reduced or not. We used the information of gene structures from four genome assemblies (BDGP6 for *D. melanogaster*, AgamP4 for *A. gambiae*, AaegL3 for *A. aegypti*, and CpipJ2 for *C. quinquefasciatus*) and the information of maker annotation of *B. antarctica* and *P. steinenii*. Among the six insects, the average intron length of *B. antarctica* (302 bp) was reported as the smallest, but that of *P. steinenii* (319 bp) was similar to that of *B. antarctica* (Fig. 1B). Despite 39 Mbp difference in genome size between *B. antarctica* and *P. steinenii*, the average length of gene regions and CDS was also similar, but the average intron number in orthologous genes was highest in *P. steinenii* (Fig. 1B).

## GO enrichment test

We identified which GO terms of 437 orthologous groups were statistically represented versus GO terms of total genes of *P. steinenii* using AgriGO [15]. AgriGO is a web-based tool for GO analysis and we tested GO terms with significant levels of p = 0.05. Complete hierarchies of GO terms for each gene were examined. Eighteen GO terms in biological process, 5 GO terms in cellular component, and 18 GO terms were identified significantly by GO enrichment analysis (Table 6). Enriched GO terms in this test proposed that the proteins associated with unfolded protein response [16] under stress conditions were developed

independently. Representative GO terms related with unfolded protein response were RNA splicing, via endonucleolytic cleavage and ligation (GO:0000394), response to unfolded protein (GO:0006986), and endoplasmic reticulum unfolded protein response (GO:0030968) in biological process.

**Table 6. GO terms were statistically overrepresented only in *P. steinenii*.**

| GO ID | GO tree | Term | number of target genes in term | number of genes in terms | p-value | FDR |
|---|---|---|---|---|---|---|
| GO:0006508 | P | proteolysis | 106 | 632 | 8.60E-13 | 2.60E-10 |
| GO:0006397 | P | mRNA processing | 32 | 120 | 6.80E-10 | 1.00E-07 |
| GO:0070054 | P | mRNA splicing, via endonucleolytic cleavage and ligation | 8 | 8 | 1.40E-09 | 1.40E-07 |
| GO:0016071 | P | mRNA metabolic process | 32 | 130 | 5.80E-09 | 4.50E-07 |
| GO:0000394 | P | RNA splicing, via endonucleolytic cleavage and ligation | 8 | 11 | 1.90E-07 | 1.10E-05 |
| GO:0006986 | P | response to unfolded protein | 6 | 7 | 1.50E-06 | 7.60E-05 |
| GO:0019538 | P | protein metabolic process | 173 | 1 506 | 2.20E-06 | 9.40E-05 |
| GO:0051789 | P | response to protein stimulus | 6 | 8 | 5.50E-06 | 0.00021 |
| GO:0006950 | P | response to stress | 50 | 330 | 8.00E-06 | 0.00027 |
| GO:0006468 | P | protein amino acid phosphorylation | 42 | 272 | 2.40E-05 | 0.00074 |
| GO:0080135 | P | regulation of cellular response to stress | 9 | 24 | 4.80E-05 | 0.0013 |
| GO:0006396 | P | RNA processing | 34 | 210 | 5.00E-05 | 0.0013 |
| GO:0051347 | P | positive regulation of transferase activity | 8 | 22 | 0.00016 | 0.0031 |
| GO:0033674 | P | positive regulation of kinase activity | 8 | 22 | 0.00016 | 0.0031 |
| GO:0045860 | P | positive regulation of protein kinase activity | 8 | 22 | 0.00016 | 0.0031 |
| GO:0034620 | P | cellular response to unfolded protein | 4 | 5 | 0.00017 | 0.0031 |
| GO:0030968 | P | endoplasmic reticulum unfolded protein response | 4 | 5 | 0.00017 | 0.0031 |
| GO:0042246 | P | tissue regeneration | 6 | 13 | 0.00024 | 0.0041 |
| GO:0031463 | C | Cul3-RING ubiquitin ligase complex | 5 | 5 | 2.90E-06 | 0.00019 |

| | | | | | | |
|---|---|---|---|---|---|---|
| GO:0031461 | C | cullin-RING ubiquitin ligase complex | 5 | 12 | 0.0014 | 0.047 |
| GO:0005789 | C | endoplasmic reticulum membrane | 11 | 55 | 0.0032 | 0.063 |
| GO:0042175 | C | nuclear envelope-endoplasmic reticulum network | 11 | 57 | 0.0042 | 0.063 |
| GO:0044432 | C | endoplasmic reticulum part | 11 | 58 | 0.0049 | 0.063 |
| | | | | | | |
| GO:0004252 | F | serine-type endopeptidase activity | 76 | 292 | 3.70E-20 | 5.50E-18 |
| GO:0004540 | F | ribonuclease activity | 30 | 54 | 1.90E-19 | 1.40E-17 |
| GO:0008236 | F | serine-type peptidase activity | 76 | 318 | 6.90E-18 | 2.50E-16 |
| GO:0017171 | F | serine hydrolase activity | 76 | 318 | 6.90E-18 | 2.50E-16 |
| GO:0004175 | F | endopeptidase activity | 84 | 416 | 5.70E-15 | 1.70E-13 |
| GO:0070011 | F | peptidase activity, acting on L-amino acid peptides | 103 | 570 | 1.60E-14 | 4.00E-13 |
| GO:0008233 | F | peptidase activity | 103 | 595 | 2.40E-13 | 5.10E-12 |
| GO:0004518 | F | nuclease activity | 30 | 102 | 1.70E-10 | 3.10E-09 |
| GO:0031072 | F | heat shock protein binding | 10 | 17 | 1.00E-07 | 1.60E-06 |
| GO:0004672 | F | protein kinase activity | 47 | 300 | 5.90E-06 | 8.70E-05 |
| GO:0008234 | F | cysteine-type peptidase activity | 15 | 59 | 3.70E-05 | 0.00049 |
| GO:0016787 | F | hydrolase activity | 171 | 1 580 | 5.00E-05 | 0.00061 |
| GO:0016773 | F | phosphotransferase activity, alcohol group as acceptor | 49 | 363 | 0.00018 | 0.002 |
| GO:0042802 | F | identical protein binding | 10 | 38 | 0.00052 | 0.0055 |
| GO:0031625 | F | ubiquitin protein ligase binding | 5 | 12 | 0.0014 | 0.014 |
| GO:0005515 | F | protein binding | 229 | 2 357 | 0.0015 | 0.014 |
| GO:0016301 | F | kinase activity | 48 | 405 | 0.0032 | 0.027 |
| GO:0003676 | F | nucleic acid binding | 144 | 1 469 | 0.0055 | 0.045 |

## Likelihood analysis of gene gain and loss

The size of gene families had been changed through evolution [17, 18]. To estimate the average gene expansion/contraction rate and to identify gene families that have undergone significant size changes, we estimated differences in the size of 15,633 orthologs using the program CAFE3.0 (www.bio.indiana.edu/~hahnlab/Software.html) [19]. The ultrametric tree of the species drawn through Timetree [20] was used for the analysis (Fig. 1C). We performed the program using $p < 0.05$, estimated birth ($\lambda$) and death ($\mu$) rates were calculated

by using the program LambdaMu with “–s” option. We calculate the number of gene gains and losses on each branch of the tree with “–t” option. Average expansion size of two Antarctic midges were relatively lower than other insects (Fig. 1C), and average expansion size of *D. melanogaster* showed the highest score among six insects. Using $p < 0.0001$ in family-wide p-values, we expect there to be approximately one significant result by chance and calculated the exact p-values for transitions over every branch. We called individual branches significant at $p < 0.005$ [21]. We could identify that 3 and 2 gene families were significantly expanded in *P. steinenii* and *B. antarctica*, respectively (Table 7).

**Table 7. Gene families were significantly expanded in Antarctic midges**

| ID | Annotation | *P. steinenii* | *B. antarctica* | 1* | 2* | *C. quinquefasciatus* | *A. aegypti* | 3* | *A. gambiae* | 4* | *D. melanogaster* | Family-wide P-values | *P. steinenii* | *B. antarctica* | 1* | 2* | *C. quinquefasciatus* | *A. aegypti* | 3* | *A. gambiae* | 4* | *D. melanogaster* |
|---|---|---|---|---|---|---|---|---|---|---|---|---|---|---|---|---|---|---|---|---|---|---|
| PS0025 | leucine rich membrane protein | 42 | 0 | 0 | 0 | 0 | 0 | 0 | 0 | 1 | 0 | 0 | 0 | 0.625 | 0.073 | 0.161 | 0.5 | 0.5 | 0.5 | 0.5 | 0.509 | 0.14 |
| PS0032 | clip-domain serine protease | 40 | 0 | 0 | 0 | 0 | 0 | 0 | 0 | 1 | 0 | 0 | 0 | 0.625 | 0.073 | 0.161 | 0.5 | 0.5 | 0.5 | 0.5 | 0.509 | 0.14 |
| PS0098 | zinc finger protein | 26 | 1 | 0 | 0 | 0 | 0 | 0 | 0 | 1 | 0 | 0 | 0 | 0.875 | 0.073 | 0.161 | 0.5 | 0.5 | 0.5 | 0.5 | 0.509 | 0.14 |
| PS0074 | serine protease | 0 | 29 | 0 | 0 | 0 | 0 | 1 | 0 | 1 | 0 | 0 | 0.625 | 0 | 0.073 | 0.161 | 0.5 | 0.5 | 0.5 | 0.5 | 0.509 | 0.14 |
| PS0114 | leucine rich repeat protein | 0 | 26 | 0 | 0 | 0 | 0 | 1 | 0 | 1 | 0 | 0 | 0.625 | 0 | 0.073 | 0.161 | 0.5 | 0.5 | 0.5 | 0.5 | 0.509 | 0.14 |

Although *B. antarctica* is notable for being freeze tolerant in its larval stages [5], the anti-freezing protein has not yet been identified from the genome, and the mechanism is unclear. In this report, we present the draft genome and annotation of the Antarctic midge, *P. steinenii*. The genome of *P. steinenii*, which is only cold tolerant, rather than freeze tolerant, in their larval stage [1, 3], will help to clarify the mechanism for freeze tolerance.

## Availability of supporting data

Supporting data are available in the GigaDB database, and the raw data were deposited in the PRJNA284858 (SRX1976250–5).

## <u>Declarations:</u>

### List of abbreviations

Gbp; giga base pairs, Mbp; mega base pairs; GO, gene ontology; EC, enzyme commission; CDS, coding sequence; SRA, short read archive


### Funding

This study was supported by Korea Polar Research Institute (grants PE16070 and PE16080).


### Authors' Contributions

SHK, HGC, HP, and SCS designed the study. SHK, WSJ, HGC collected the samples and performed the experiments, S.C.S, H.P, and J.H.P analyzed the data. All authors participated in the writing of the manuscript.

## Figure Legends

**Fig. 1. Genome-wide analysis of protein-coding genes in *P. steinenii*.** **(A)** Venn diagram displaying the overlap in orthologous genes in six insect species. **(B)** The statistics of gene structure of the six insects. **(C)** Lineage-specific gene gain and loss among the 6 insects. The numbers in boxes are identifiers for internal branches of the phylogeny. Numbers on each branch denote the number of expansion, remain, and decress. AE denotes average expansion.

## References


[1] Convey P, Block W. Antarctic Diptera: ecology, physiology and distribution. Eur J Entomol. 1996;93:1–14.

[2] Edwards M, Usher MB. The winged Antarctic midge *Parochlus steinenii* (Gerke)(Diptera: Chironomidae) in the South Shetland Islands. Biol J Linnean Soc. 1985;26(1):83–93.

[3] Shimada K, Ohyama Y, Pan C. Cold-hardiness of the Antarctic winged midge *Parochlus steinenii* during the active season at King George Island. Polar Biol. 1991;11(5):311–4.

[4] Gnerre S, MacCallum I, Przybylski D, Ribeiro FJ, Burton JN, Walker BJ, et al. High-quality draft assemblies of mammalian genomes from massively parallel sequence data. Proc Natl Acad Sci. 2011;108(4):1513–8.

[5] Kelley JL, Peyton JT, Fiston-Lavier AS, Teets NM, Yee MC, Johnston JS, et al. Compact genome of the Antarctic midge is likely an adaptation to an extreme environment. Nature Commun. 2014;5.

[6] Cantarel BL, Korf I, Robb SM, Parra G, Ross E, Moore B, et al. MAKER: an easy-to-use annotation pipeline designed for emerging model organism genomes. Genome Res. 2008;18(1):188–96.

[7] Tarailo-Graovac M, Chen N. Using RepeatMasker to identify repetitive elements in genomic sequences. Curr Protoc Bioinformatics. 2009;4.10. 11-14.10. 14.

[8] Korf I. Gene finding in novel genomes. BMC Bioinformatics. 2004;5(1):1.

[9] Conesa A, Götz S, García-Gómez JM, Terol J, Talón M, Robles M. Blast2GO: a universal tool for annotation, visualization and analysis in functional genomics research. Bioinformatics. 2005;21(18):3674–6.

[10] Bao Z, Eddy SR. Automated de novo identification of repeat sequence families in sequenced genomes. Genome Res. 2002;12(8):1269–76.

[11] Price AL, Jones NC, Pevzner PA. De novo identification of repeat families in large genomes. Bioinformatics. 2005;21Suppl 1 :i351–8.

[12] Benson G. Tandem repeats finder: a program to analyze DNA sequences. Nucleic Acids Res. 1999;27(2):573.

[13] Lowe TM, Eddy SR. tRNAscan-SE: a program for improved detection of transfer RNA genes in genomic sequence. Nucleic Acids Res. 1997;25(5):955–64.

[14] Li L, Stoeckert CJ, Roos DS. OrthoMCL: identification of ortholog groups for eukaryotic genomes. Genome Res.2003;13(9):2178–89.

[15] Du Z, Zhou X, Ling Y, Zhang Z, Su Z. agriGO: a GO analysis toolkit for the agricultural community. Nucleic Acids Res. 2010:gkq310.

[16] Chen Y, Brandizzi F. IRE1: ER stress sensor and cell fate executor. Trends Cell Biol. 2013;23(11):547–55.

[17] Lynch M, Conery JS. The evolutionary fate and consequences of duplicate genes. Science. 2000;290(5494):1151–5.

[18] Hahn MW, De Bie T, Stajich JE, Nguyen C, Cristianini N. Estimating the tempo and mode of gene family evolution from comparative genomic data. Genome Res. 2005;15(8):1153–60.

[19] De Bie T, Cristianini N, Demuth JP, Hahn MW. CAFE: a computational tool for the study of gene family evolution. Bioinformatics. 2006;22(10):1269–71.

[20] Hedges SB, Dudley J, Kumar S. TimeTree: a public knowledge-base of divergence times among organisms. Bioinformatics. 2006;22(23):2971–2.

[21] Hahn MW, Han MV, Han S-G. Gene family evolution across 12 Drosophila genomes. PLoS Genet. 2007;3(11):e197.

Figure
Click here to download Figure Figure01.jpg

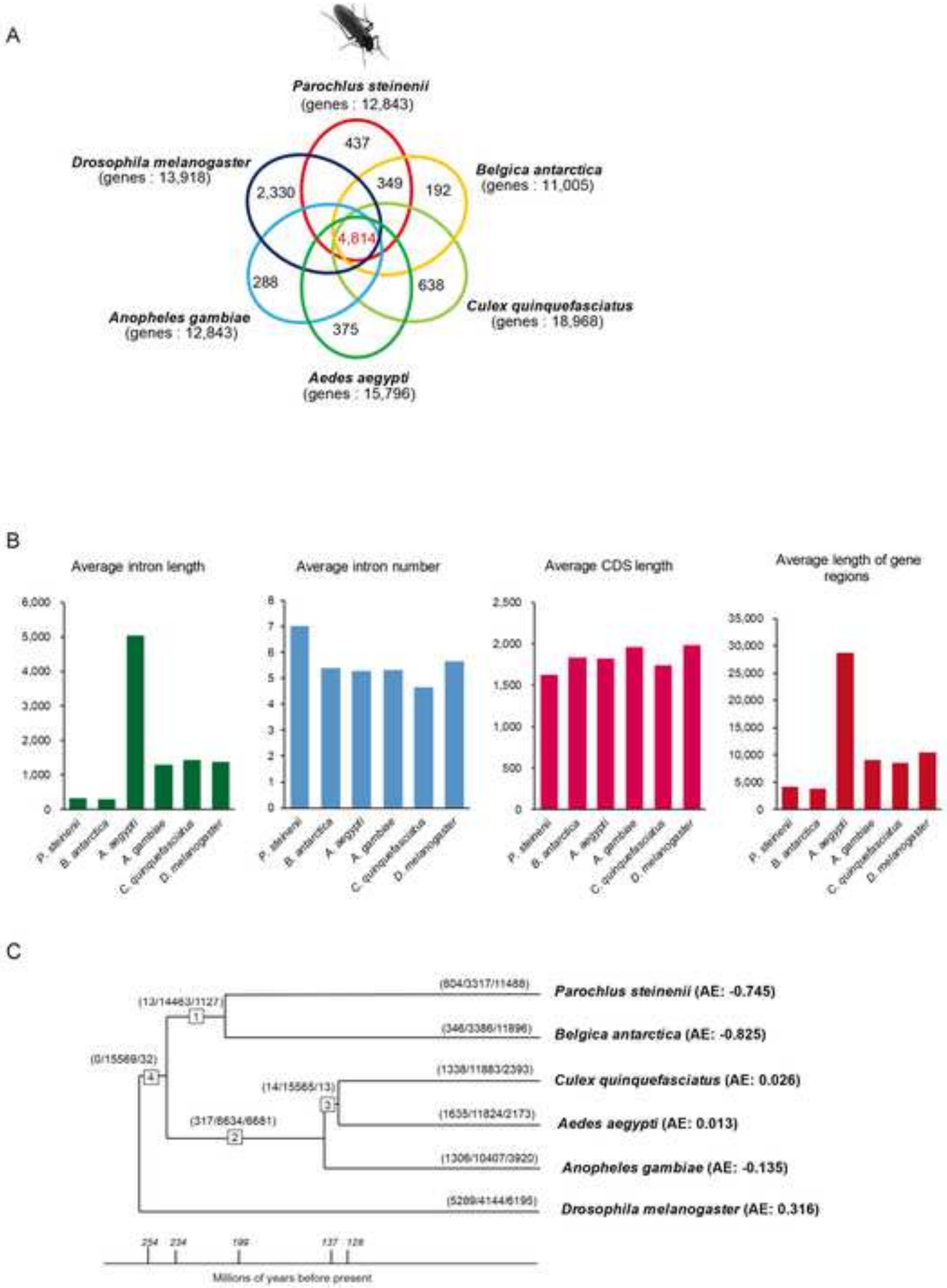

Personal Cover
Click here to download Personal Cover
Parochlus_cover_letter.docx

July 29, 2016

Dear Editor:

We wish to submit a new manuscript entitled, "**Genome sequencing of the winged midge, *Parochlus steinenii*, from the Antarctic Peninsula**", to be considered for publication in *GigaScience.*

In the Antarctic, only two species of Chironomidae occur naturally: the wingless midge *Belgica antarctica,* and the winged midge *Parochlus steinenii*. *B. antarctica* is notable for its tolerance to freezing, and its compact genome is thought to be the result of adaptation to an extreme environment. Despite this, an anti-freezing protein has not yet been identified in the genome, and the mechanism of freeze tolerance is unclear.

In this study, we present the annotated, draft genome of the Antarctic midge, *P. steinenii*. *P. steinenii* is cold tolerant but not freeze tolerant in the larval stage, so its genome will help to clarify the mechanism for freeze tolerance when compared with that of *B. antarctica*.

I confirm that all authors have approved the manuscript for submission, and the content of the manuscript has not been published, or submitted for publication, elsewhere.

Please address all correspondence concerning this manuscript to me, at ssc@kopri.re.kr.

Thank you for considering our manuscript.

Sincerely,

Seung Chul Shin
Division of Polar Life Sciences
Korea Polar Research Institute
26 Songdomirae-ro, Yeonsu-gu, Incheon 21990
South Korea
