# Supplementary material for: Genome sequencing of the winged midge, Parochlus steinenii, from the Antarctic Peninsula
Source: Gigascience. 2017 Feb 24;6(3):1–8. doi: 10.1093/gigascience/giw009 (PMC5467013; doi:10.1093/gigascience/giw009)
Supplement: Response_to_Reviewers_Original_Submission.pdf [file giw009_Response_to_Reviewers_Original_Submission.pdf]

Dear Editor:

We are pleased to have an opportunity to revise our manuscript, entitled, “Genome sequencing of the winged midge, Parochlus steinenii, from the Antarctic Peninsula". In revising the paper, we carefully considered your comments and suggestions, as well as those offered by the reviewers.
As instructed, we explained how we revised this manuscript based on the comments and recommendations. We greatly appreciate the time and effort put forth to provide us insightful guidance.
The revision includes a number of changes:
- We changed the background information in the abstract, as requested by reviewers.
- We added a more detailed description of methods and parameters used.
- We clarified portions of the methodology.
- We added the results of the BUSCO and CEGMA analyses.

In rebuttal letter, we offer detailed responses to your comments, as well as those of the reviewers.
We hope that our revisions improved the quality of this manuscript and thank you again for your consideration of our manuscript.

Sincerely,
Seung Chul Shin

Reviewer reports:

Reviewer #1: This paper describes the genome assembly and annotation of the winged midge, Parochlus steinenii. This species is of particular interest, as it co-occurs in Antarctica with another midge species, Belgica antarctica, but is cold-tolerant; comparative analyses of these two genomes may yield insights into the origins of freeze-tolerance in Belgica antarctica. The data generated and the analyses performed are useful, and should be valuable to the insect comparative genomics community. However, there are a number of uncertainties with the manuscript. Specifically, many details of the analyses are left out, which will make it difficult for others to 1) understand and 2) replicate the analyses performed. It is possible that these details are contained within the 'Supporting data' in the GigaDB database, but I do not have access to these records, and there are no mentions of the Supporting data within the manuscript, except under 'Availability of supporting data'.

Specific Comments:

Abstract - Background
l. 28: "with a compact genome as a result of adaptation to an extreme environment": As far as I know, there are no studies yet that have determined that B. antarctica's small genome size is a result of the extreme environment this insect lives in (the 2014 genome paper doesn't go that far). Please soften the language, or provide a citation that demonstrates a causal relationshiop.
> We agree with the reviewer’s comments and have rewritten the sentence as follows:
“B. antarctica is an extremophile with unusual adaptations. The larvae of B. antarctica are desiccation- and freeze-tolerant and the adults are wingless.”

l. 31: change "are cold, but not freeze, tolerant" to "are cold- but not freeze-tolerant,"
> We have changed “are cold, but not freeze, tolerant” to “are cold-tolerant but not freeze-tolerant” in the revised manuscript.

Abstract - Conclusions
l. 44: Please change "cold, but not freeze, tolerant" to "cold- but not freeze- tolerant"
> We have rewritten the conclusions as follows:
“We present an annotated draft genome of the Antarctic midge, P. steinenii. The genomes of P. steinenii and B. antarctica will aid in the elucidation of evolution under harsh environments and provide new resources for functional genomic analyses of the order Diptera.”

Data description - Sequencing
l. 49. How many individuals? Did you determine the sex, or was it a mixed collection? If there were too many to count, did you weigh them? What life history stage?
> We have added the description in the revised manuscript as follows:
"Twenty adults were used for genome sequencing, regardless of gender."

l. 61: What life history stage? How many insects?
> We have changed "whole body of P. steinenii using the Qiagen kit" to "whole body of 10 adults in three different groups using the RNeasy mini kit (Qiagen, Valencia, CA, USA)"

l. 61: Which Qiagen kit was used?
> We have changed "Qiagen kit" to "RNeasy mini kit" in the manuscript.

l. 67: Which Fastx program was used?
> We have changed "using the FASTX-Toolkit" to "using the fastq_qulity_trimmer in the FASTX-Toolkit" in the revised manuscript.

l. 69: I don't understand what you mean by "data from paired-end trimmed reads with 14 gigabase pairs (Gbp) were obtained". I couldn't find a table legend that explained this, either.
Table 1:
- I couldn't find a table legend.
> We have changed "data from paired-end trimmed reads with 14 gigabase pairs (Gbp) were obtained (Table1)" to "yields after quality trimming for the fragment library totaled 14.8 gigabase pairs (Gbp)."

We have added a table legend as follows:
"Tree-type libraries were constructed in this study. A PE400 library was constructed as a fragment library for ALLPATHS-LG. Mate-pair (MP3K and MP5K) libraries were also constructed for ALLPATHS-LG assembly. Three PE300 libraries (PE300A, PE300B, and PE300C) were constructed from RNA for gene annotation."

Additionally, we have added the library name from the table into the manuscript as follows:
"One was a fragment library, which was a paired-end type with an insert size of 400 bp (PE400), whereas others were jumping libraries, which were mate-pair types with insert sizes of 3 kbp (MP3K) and 5 kbp (MP5K)."
"Three paired-end libraries with an insert size of 300 bp (PE300) were constructed using the TruSeq Stranded mRNA Library Prep Kit (Illumina, San Diego, CA, USA)"

- the column 'Read lengths' doesn't make sense to me - is this the combined length of all reads?
> We have changed "Read lengths" to "Total read lengths"

Data description - Genome assembly
l. 78: "the fragment library should be designed to overlap": Do you mean that the reads from paired-end library overlapped, and were thus combined to generate one longer read?
> Yes. We have rewritten the sentence as follows:
"For better assembly in ALLPATHS-LG, a larger k-mer size was used with one longer read generated from the paired-end library [4]. As a result, the paired-end reads from the fragment library were designed to overlap, and the insert size of the paired-end library was slightly less than twice the read size [4]. In this assembly, 93.8% of the paired-end reads from the fragment library overlapped and merged into one longer read."

l. 79: I don't understand what this means: "In this assembly, 93.8% of the fragment library was full".
> We have rewritten the sentence as follows:
"93.8% of paired-end reads from the fragment library overlapped and merged into one longer read."

l. 81: "The resulting assembly had a total size of 137 Mb" - In table 2, you list 130.6 Mb for the contigs and 138 Mb for the scaffolds. Where do you get the number 137 Mb from?
> We have corrected 137 Mb with 138 Mb.

l. 83: How did you calculate the coverage?
> We estimated contig coverage by total read lengths from the fragment library, but we used the coverage in the assembly report from ALLPATHS-LG in the revised manuscript.
Thus, we have changed "revealed contig coverage of approximately 108.5 ×" to "revealed contig coverage of approximately 89 × total read lengths from the fragment library."

Data description - Gene annotation
l. 93: "For proper gene annotation, RNA and protein evidence alignment were used".
- Which RNAs were used? At what step in the Maker program?
> For RNA evidence, we extracted total RNA from the whole body of adults, sequenced, and assembled the resulting reads into contigs. The resulting contigs were used for the MAKER2 annotation pipeline to find the best gene model using RNA evidence with the alignment results of proteins. We have clarified the RNA evidence in the revised manuscript as shown in the response to the next comment.

- You list the proteins that were used to train Maker in the next paragraph (l. 99-104). Please list them here, instead.
> We have rewritten the paragraph as follows:
"To find the best possible gene model for the given region, RNA and protein evidence alignment were considered in MAKER2 [17]. Transcriptome assembly results were used for RNA evidence, the paired-end reads resulting from mRNA of the whole body of adults were trimmed using the fastq_qulity_trimmer in the FASTX-Toolkit (Ver. 0.0.11) (http://hannonlab.cshl.edu/fastx_toolkit) with the parameters -t 30, -l 80, and -Q 33, and they were assembled with CLC Genomics Workbench (Ver. 8.0.0) with default parameters. In all, 68,392 contigs with an N50 contig size of 435 bp and an average contig size of 407 bp, were generated and used for RNA evidence. Protein sequences from six species, given in NCBI reference sequences, were used for protein evidence—Drosophila melanogaster (fruit fly, GCF_000001215.4), Ceratitis capitata (Mediterranean fruit fly, NC_000857.1), Bactrocera dorsalis (oriental fruit fly, NC_008748.1), Anopheles gambiae (African malaria mosquito, NZ_AAAB00000000.1), Aedes aegypti (yellow fever mosquito, AAGE00000000.2), and Culex quinquefasciatus (southern house mosquito, AAWU01000000). Alignment of transcriptome assembly with BLASTn and alignment of homologous protein information from tBLASTx were considered as evidence for annotation."

l. 94: What ESTs were used?
> We used the transcriptome assembly as RNA evidence instead of EST. Thus, we have changed "expressed sequencing tags" to "transcriptome assembly" in the revised manuscript.

l. 96: What transcriptome assembly? Does this line describe the assembly of the RNA data that were generated? Were reads trimmed prior to the assembly? This needs more detail.
> To clarify the transcriptome assembly in the manuscript, we have added the details for transcriptome assembly as follows
"To find the best possible gene model for the given region, RNA and protein evidence alignment were considered in MAKER2 [17]. Transcriptome assembly results were used for RNA evidence, the paired-end reads resulting from mRNA of the whole body of adults were trimmed using the fastq_qulity_trimmer in the FASTX-Toolkit (Ver. 0.0.11) (http://hannonlab.cshl.edu/fastx_toolkit) with the parameters -t 30, -l 80, and -Q 33, and they were assembled with CLC Genomics Workbench (Ver. 8.0.0) with default parameters. In all, 68,392 contigs with an N50 contig size of 435 bp and an average contig size of 407 bp, were generated and used for RNA evidence."

l. 111: "This was annotated with the BLASTp results and InterproScan [9]." I'm confused by this sentence - what is "This"? which BLASTp results - is this output from the Blast2Go program, or another analysis? Also, are the InterproScan results part of the Blast2Go analysis?

> We have changed “gene ontology (GO) classified 10,801 (80.2%) of the predicted genes to a function. This was annotated with the BLASTp results and InterproScan [9].” with “gene ontology (GO) classified 10,801 (80.2%) of the predicted genes to a function using the BLASTp and InterproScan results [9].”

Data description - Gene annotation for B. antarctica
l. 125 - are the six other species used for protein evidence the same that are listed on l. 99-104?
> We have changed “We matched proteins from P. steinenii to those from six species for protein evidence.” to “We used the same protein sequence from the six species used for gene annotation in P. steinenii and predicted proteins of P. steinenii for protein evidence.”

l. 143: How did the methods for repeat analysis differ between this paper and the B. antarctica genome paper (cited in [5])?
> In the case of repeat analysis for B. antarctica, RepeatMasker and the T-lex2 de novo pipeline were used for repeat analysis. We only used RepeatMasker for repeat annotation. Thus, it might be improper to compare the results of repeat analysis directly.

Table 4 - This could probably be moved to a supplement, or condensed.
> As suggested by reviewer, we have made Table 4 a Supplementary Table.

Data description - Ortholog analysis
Table 5 - This should be moved to a supplement, or condensed. Also, is there supporting data that lists what genes are in which group? It is interesting that D. melanogaster has so many unique proteins, compared to the other 5 species. Can you speculate why?
> We have made Table 5 a Supplementary Table and added the number of unique orthologous groups of six species to Figure 1a. D. melanogaster belongs to the suborder Brachycera and the other five species belong to a different suborder, Nematocera, in Diptera. This might be the reason why D. melanogaster showed so many unique orthologous groups.

Data description - Gene structure of Orthologous groups
l. 174 - Are you using the genome size for B. antarctica that was calculated by flow cytometry for this comparison? Perhaps you should use the range calculated from genome sequencing, instead, since this is how you are estimating the P. steinenii genome size.
> We used the assembled genome size for B. antarctica and for P. steinenii. Thus, we have changed “Despite 39 Mbp difference in genome size ……” to “Despite approximately 50 Mbp difference in the assembled genome size between B. antarctica and P. steinenii, ……’.

Data description - GO enrichment test
l. 180: "statistically represented" - do you mean over-represented?
> We have changed from “represented” to “overrepresented.”

l. 180: "437 orthologous groups" - please change to "437 orthologous groups that are unique to P. steinenii"
> We have changed from "437 orthologous groups" to "437 groups that were unique to P. steinenii."

l. 181: AgriGO has several analysis tools. Which one did you use, with what parameters?
> We have rewritten the sentence to clarify the methods as follows:
“AgriGO is a web-based tool for GO analysis, we selected “Fisher’s exact test” for the statistical test method and selected “Hochberg FDR” as the multiple test adjustment method. GO terms were tested with a significance level of $p < 0.05$.”

l. 182: "significant levels of $p = 0.05$" - do you mean "significance levels of $p < 0.05$"?
> We have corrected “=” with “<.”

l. 185: Can you 1) explain what an unfolded protein response is, and how this may be biologically

interesting for P. steinenii, and 2) elaborate on why you think an enrichment for genes associated with 'unfolded protein response under stress conditions' in the orthomcl groups that are unique to P. steinenii implies that they evolved independently? Finally, why did you only single out these categories, when many more were enriched for genes in orthomcl groups unique to P. steinenii?
> We have added the description to the unfolded protein response. Because 14 GO terms among 26 GO term were associated with the UPR in the GO hierarchy, we singled out these categories as representative. It is hard to explain how they evolved independently. We have rewritten the section as follows:
“It is noteworthy that 14 GO terms among 26 GO terms in biological processes were associated with the unfolded protein response (UPR). The UPR is a stress response that occurs in the lumen of the endoplasmic reticulum (ER) [22]. When unfolded or misfolded proteins were accumulated in the ER lumen under stress conditions, the UPR is activated to improve protein folding by increasing the production of chaperones [22].”

Data description - Likelihood analysis of gene gain and loss
l. 198 - The URL doesn't work.
> We have deleted the URL and kept only a reference for café3.0.

l. –99 - How did you generate the tree? From what datasets?
> To clarify the methods used to generate the tree, we have added a section to the manuscript as follows:
“we estimated differences in the size of 15,633 orthologs using the program CAFE3.0 [25]. A Newick description of a rooted and bifurcating phylogenetic tree was needed for this analysis. Therefore, we performed phylogenetic analyses among six insects with the protein-coding gene in the orthologous groups. We selected 4,814 orthologous gene sets from the orthologous groups from OrthoMCL using the reciprocal best BLASTP hit criteria. Protein-coding gene sequences were aligned using PRANK (Ver. 130820) under a codon model with the “-DNA and –codon” option [26], poor alignment sites were eliminated using Gblock (Ver. 0.91) under a codon model with the “-t = c” option [27], and the remaining alignment regions were concatenated to be used in the phylogenetic analyses. The phylogenetic tree was constructed using the neighbor-joining method [28] in the MEGA version 6 program [29]. With the resulting phylogenetic tree, we prepared the ultrametric tree of the species, including branch lengths in units of time through TimeTree [30], for the analysis (Figure 1C).”

l. 218 - Are you going to deposit the genome assembly in a public repository?
> If the genome assembly is suitable to be deposited in the GigaDB, we do not plan to deposit it in other public repositories.

General
- I couldn't find any Table legends. These are essential, as the tables by themselves are not descriptive enough.
> We have added legends to all tables.

- It is not clear what supporting data are in the GigaDB database - is there a way to make this obvious to the reader in the manuscript?
> We have added a better description for supporting data as follows:
“Supporting data (sequence files for CDS, protein, transcript, and the draft genome, and the general feature format for genes and repeats) are available in the GigaDB database, and the raw data were deposited in the PRJNA284858 (SRX1976250–5).”

Reviewer #2: A few comments:
- Table 2: Even though scaffolding greatly improved the assembly there is still a great number of scaffolds (>4,000) and a relatively low scaffold N50, compared to the genome size of this midge (~138 Mbp). In addition, I would say that this unexpected given the amount of sequencing data generated for this insect, which resulted in >100x average contig coverage. I would suggest that the authors comment on it and mention some probable causes for this (e.g. increased repeat content, increased heterozygosity, no mate-pair libraries with an insert of >5 Kbp?).

> Approximately 89 × total reads lengths from the fragment library was used and two mate-pair libraries were used in this assembly. A total of 57.2% of the 3-kb jumping library and 33.1% of the 5-kb jumping library were used, and 9.6 kb of the N50 contig size was increased to 157kb of the N50 scaffold size. More jumping libraries and long jumping libraries might improve this assembly and another fragment library might improve it by increasing the randomness of reads in the library.

We have added the sentences as follows:
"A total of 57.2% of the 3-kb jumping library and 33.1% of the 5-kb jumping library were used and 9.6 kb of the N50 contig size was increased to 157 kb of the N50 scaffold size. If more jumping libraries or long jumping libraries (the insert size was larger than 20 kbp) were used, the scaffolding might improve the assembly."

- Lines 73-83: While the authors mention all the tools and parameters used for genome assembly, there is no mention about the tool they used for scaffolding. I think it would be nice to add this important information, especially since scaffolding contributes to a significant improvement of the assembly.
> Assembly was performed using ALLPATHS-LG. This assembler linked the contigs into scaffolds with two mate-pair libraries. A total of 57.2% of the 3kb jumping library and 33.1% of the 5kb jumping library were used. Thus, we did not perform additional scaffolding.

- Line 92: Why did you only use SNAP for gene prediction? Augustus is known to perform better and can be run from the MAKER pipeline.
> Augustus showed better performance than SNAP in ab initio predictions, but in the MAKER pipeline, SNAP and Augustus showed similar results in evidence-based annotation (Holt et al., 2011; MAKER2: an annotation pipeline and genome-database management tool for second-generation genome projects).

- The authors haven't performed an evaluation of their genome assembly or their predicted gene set. Such evaluations are usually done by tools such as BUSCO [Simao et al. 2015], that search for conserved genes in the assembly/gene set.
> To assess the annotated gene set and genome assembly, we ran BUSCO and CEGMA analyses and have added the results to the manuscript as follows:
"The assembled genome size was similar to the predicted genome size (143.8 Mb). We also validated this assembly using CEGMA [7] and BUSCO [8]. CEGMA evaluation showed that gene completeness of this assembly was 85.08% and BUSCO analysis using arthropod databases showed 67% completeness (Tables 3 and 4). If partially matched genes were considered, 92.34% and 89.6% of the genes were identified in CEGMA and BUSCO, respectively (Tables 3 and 4)."
"To assess the annotated gene set, we ran a BUSCO analysis with the "-m OGS" options for gene set completeness and identified 70.7% genes considered to be complete with the expanded gene set, and 16.5% of the gene set was classified as missed [8]."

- Table 5 is unnecessarily long and complicated. First of all, I think that not all the different combinations are necessary to show. I would only include the largest groups and also the most biologically important (certainly no more than 10 groups). I would also suggest that the authors find descriptive names of each group, such as "P. steinenii-specific", or Antarctic midge-specific, or mosquito-specific. I find group names such as "ABC", "ABCDF", "BCEF" to not be human-readable. Last, instead of showing numbers of orthologous groups it would be more meaningful to show number of genes (and maybe show how many of them are transcribed).
> As suggested by this reviewer, we have made Table 5 a Supplementary Table and added the number of unique groups in the six species to Figure 1a.

Some more, minor comments:

- Line 33: The sentence "In this study..." is isolated does not say much. I would suggest to either delete it, or develop it to something more informative.

The ms focused on "reproducibility of analyses",
while more on the biology of this midge would make the story more exciting.
> We have deleted the sentence “In this study….” on line 33.
We have rewritten this sentence as follows:
“Differences in adaptations in the Antarctic midges are interesting in terms of evolutionary processes under an extreme environment.”

A few comments:

- Table 1: the first two lines refer to libraries "PE400trim" and "PE400". It's not clear to me if these two libraries are different or the same (with PE400tim simply being the trimmed PE400 library). The authors should clarify this.
> We have removed the row “PE400trim” and added a legend to Table 1 as follows:
“Tree-type libraries were constructed in this study. A PE400 library was constructed as a fragment library for ALLPATH-LG. Mate-pair (MP) libraries was also constructed for ALLPATH-LG assembly. Three PE300 libraries (PE300A, PE300B, and PE300C) were constructed from RNA for gene annotation.”

- Table 1: Is the column named "Read lengths" showing total read lengths? If so, please rename it.
> We have changed “read lengths” to “total read lengths.”

- Line 180: The authors refer to 437 orthologous groups but it is not clear what these are. Are they the P. steinenii-specific groups? If so, it should be clearly mentioned in the sentence to avoid confusion.
> We have changed "437 orthologous groups" to "437 groups specific in P. steinenii genes were not identified in any other species."

- Table 6: I'm not sure what the columns "number of target genes in term" and "number of genes in terms" mean. Is the former representing the number of genes with a GO term in the species-specific orthologous groups, while the latter represents the same number in the whole gene set? You should make the descriptions more clear.
> We have changed “number of target genes in term” to “the number of genes with GO terms in the P. steinenii-specific groups” and changed “number of gene in terms” to “the number of genes with GO terms in P. steinenii’s entire gene set.”

- Some typos:
- line 90: "...using MAKER2..." --> "using the MAKER2..."
> We have changed "...using MAKER2..." to "using the MAKER..."

- lines 119-124: Please rephrase the sentence "For RNA evidence...". The first part of the sentence (up to "pipe lines") does not make sense.
> We have deleted the sentence “For RNA evidence…… ……pipe lines” and added the sentence “The resulting contigs were aligned to the genome sequence of B. antarctica with BLASTn in MAKER2 for RNA evidence” as follows:
“The reads in various experimental conditions with B. antarctica (SRR566981, SRR567289, SRR567164~7, and SRR567169~71) were downloaded from SRA databases in NCBI and we assembled the reads into 38,017 contigs with an N50 contig size of 1,799 bp and an average contig size of 913 bp through CLC Genomics Workbench (Ver. 8.0.0). The resulting contigs were aligned to the genome sequence of B. antarctica with BLASTn in MAKER2 for RNA evidence.”

- line 168: "...of orthologous gene..." --> "of orthologous genes"
> We have corrected “gene” with “genes” in line 204.

- line 184: Is there something missing in "...and 18 GO terms were identified..."?
> We have added “in molecular functions” in line 223 as follows:
“…18 GO terms in molecular functions were…”

- Table 6: in the title of the table delete "were".
> We have deleted “were”

- line 195: The sentence "The size..." is not informative at all. I suggest you merge it with the next one.
> We have rewritten the sentence as follows:
“To estimate the average gene expansion/contraction rate and to identify gene families that have undergone significant size changes through evolution”

- lines 199-200: "We performed the program" --> "We ran the program".
> We have changed “performed” to “ran” in line 257

- line 205: delete "there"
> We have deleted the word “there.”

Reviewer #3: The article describes the genome assembly of the winged midge Parochlus steineii. There are comparisons to other insect genome assemblies. The genome assembly will provide a useful resource for comparative genome studies. My comments and concerns are listed below.

There are statements in the manuscript that are factually incorrect and must be remedied. The statement that B. antarctica "adults lose their wings" (line 29) is an inaccurate description of the adult wing status. The adults never have wings. Also, the genome assembly of B. antarctica proposes that the small genome is likely due to adaptation to cold environment, however, given that there is no comparative data or study of adaptation and genome size, there is not conclusive evidence that the small genome is itself adaptive.
> We have rewritten the sentence as follows:
“B. antarctica is an extremophile with unusual adaptations. The larvae of B. antarctica are desiccation- and freeze-tolerant and the adults are wingless.”

The concluding sentences of the manuscript state that the mechanisms of freeze tolerance are unknown in B. antarctica. The mechanisms are known and have been explored extensively (see 1979 paper http://onlinelibrary.wiley.com/doi/10.1111/j.1365-3032.1979.tb00171.x/abstract and http://www.ncbi.nlm.nih.gov/pubmed/16424090).
> We have rewritten the sentence as follows:
“We present an annotated draft genome of the Antarctic midge, P. steinenii. The genomes of P. steinenii and B. antarctica will aid in the elucidation of evolution under harsh environments and provide new resources for functional genomic analyses of the order Diptera.”

How many individuals were used in the genome assembly?
> Twenty adults were used for genome sequencing, regardless of gender. Thus, we have added this sentence to the revised manuscript as follows:
“Twenty adults were used for genome sequencing, regardless of gender. Genomic DNA was extracted using a DNeasy Tissue Kit (Qiagen, Valencia, CA, USA).”

What is the k-mer analysis estimate of genome size and heterozygosity?
> We have estimated the genome size and heterozygosity using the kmer analysis and added it to the revised manuscript as follows:
“Before assembly, we estimated the genome size and heterozygosity using a kmer analysis with sequencing reads. The software Jellyfish (Ver 1.1.10) [5] and GenomeScope (http://qb.cshl.edu/genomescope/) [6] were used. The 17-mers were counted in the reads from the PE400 library and the resulting histogram of 17-mers occurrence was used as a query for GenomeScope [6]. The estimated genome size was 143.8 Mb and the estimated heterozygosity was 0.613%.”

Table 2: Later in the manuscript there is a re-analysis of the B. antarctica genome for direct comparison - that data should be presented in Table 2 instead of quoting from the previous paper.

> We have added the results from the re-analysis of the B. antarctica genome to Table 2.

An additional estimate of genome size - flow cytometry, k-mer analysis, etc - would allow the actual comparison of absolute genome size between the species. The sum of scaffolds in the B. antarctica paper was ~10Mb smaller than the flow cytometry estimated size.
> We have added the estimate genome size as an additional estimate to the manuscript and we have compared the gene structure based on the assembled genome size.

The methods for the ortology analysis are missing (lines 125-127).
> This ortholog analysis was the same analysis as in the next paragraph. Thus, we have removed for clarity.

The authors should run BUSCO to present results on the putative completeness of the genome assembly.
> To assess the annotated gene set and genome assembly, we ran BUSCO and CEGMA analysis and have added the results to the revised manuscript as follows:
"The assembled genome size was similar to the predicted genome size (143.8 Mb). We also validated this assembly using CEGMA [7] and BUSCO [8]. CEGMA evaluation showed that gene completeness of this assembly was 85.08% and BUSCO analysis using arthropod databases showed 67% completeness (Tables 3 and 4). If partially matched genes were considered, 92.34% and 89.6% of the genes were identified in CEGMA and BUSCO, respectively (Tables 3 and 4)."
"To assess the annotated gene set, we ran a BUSCO analysis with the "-m OGS" options for gene set completeness and identified 70.7% genes considered to be complete with the expanded gene set, and 16.5% of the gene set was classified as missed [8]."

Was the repeat content re-estimated for the B. antarctica assembly?
> The statistics of repeats of B. antarctica are quoted from a previously published paper (Kelley et al, 2014). Thus, we have replaced the data from a previously reported paper with the results of the repeat analysis for the B. antarctica from this study.

GO enrichment test (section starting on line 179) is missing crucial information that the 437 orthologous groups are those genes that are unique to P. steinenii. Please note that they are not orthologs if they are unique to P. steinenii. In this analysis, how many genes had no term? What is the correction for genes belonging to multiple GO levels in the GO enrichment analysis?
> We have added more description to the methods for the GO enrichment test and have made corrections as follows:
"We identified which GO terms of the 437 groups that were unique to P. steinenii were statistically overrepresented versus GO terms of all genes of P. steinenii using AgriGO [21]. A total of 1,352 genes comprised 437 groups and therein were 717 genes with GO terms. AgriGO is a web-based tool for GO analysis, we selected "Fisher's exact test" for the statistical test method and selected "Hochberg FDR" as the multiple test adjustment method. GO terms were tested with a significance level of $p < 0.05$."

What dataset was used for Timetree?
> TimeTree looks for two queried species in the searchable tree of life scaled by time and produces the time estimate available. We used the estimated divergence time from TimeTree.

What is the estimated divergence time of the two species?
> The estimated divergence time of the two species is 199 millions years and the estimated divergence time was added in the legend to Figure 1c.
"…Numbers on each branch denote the number of gained, lost, and stable genes. AE denotes the average expansion. The number below each corresponding node denotes the estimated divergence time based on TimeTree."

Minor comments:
Table 1: why are both the pre-and post-trimmed datasets shown for the PE400 data but not the other

datasets? Why are three lines shown for the RNAseq PE300 data? Are the three lines from the same library? If so, the amounts can be summed and presented on one line, if not, the differences need to be explained in the table and in the text.
> We have removed the row "PE400trim" and have added a legend to Table 1 as follows:
"Tree-type libraries were constructed in this study. A PE400 library was constructed as a fragment library for ALLPATHS-LG. Mate-pair libraries (MP3K and MP5K) were also constructed for ALLPATHS-LG assembly. Three PE300 libraries (PE300A, PE300B, and PE300C) were constructed from RNA for gene annotation."
We have added more description to the methods in the revised manuscript as follows:
", total RNA was extracted from the whole body of 10 adults in three different groups using the RNeasy mini kit (Qiagen, Valencia, CA, USA), according to the manufacturer's instructions."

Table 2 and the text are not consistent with regards to the reported size of the genome.
> We have corrected "137 Mb" with "138 Mb" in the manuscript.

What does "93.8% of the fragment library was full" mean? It is not clear in the text.
> We have rewritten the sentence as follows:
"93.8% of the paired-end reads from the fragment library overlapped and merged into one longer read."

Several of the tables should be included as supplementary tables and not in the main text (Table 4 and Table 5, for example).
> As the reviewer suggested, we have moved the Tables 4 and Table 5 and made them as Supplementary Tables.

Table 7 is unclear, what are the 1*, 2* etc?

> We have added this to the legend of the Table, which is now Table S3.
"* The numbers are identifiers for internal branches of the phylogeny (Figure 1C)"

The use of "expressed sequence tags" to describe RNAseq is incorrect.
> We have changed "expressed sequencing tags" to "RNA evidence" on line 62 and changed "expressed sequencing tags" to "transcriptome assembly" on line 165.

The sentence on lines 68-69 does not make sense.
> We have rewritten the sentence as follows:
"Finally, yields after quality trimming for the fragment library totaled 14.8 gigabase pairs (Gbp)."

Blast2Go is Blast2GO. Similarly, maker should be capitalized.
> We have corrected "Blast2Go" with "Blast2GO".

Reviewer #4: The authors describe their sequencing of this midge genome and a pretty typical set of metrics of evaluating it, but not much more. I understand this is acceptable for a Data Note, so instead have focused this review on making the work more readable and interpretable.

L28. In the Background of the Abstract, the authors says that "B. antartica has unusual characteristics with a compact genome as a result of adaptation to an extreme environment". I don't think there is any evidence that the compactness of that genome has anything to do with the extreme Antarctic environment, it could just be a coincidence. Perhaps other members of that genus or that lineage of midges has similarly tiny genomes, and even if they don't, one would require study of many independent origins of cold-hardiness to say small genomes result from adaptation to extreme environments.
> We have rewritten the sentence as follows:
"B. antarctica is an extremophile with unusual adaptations. The larvae of B. antarctica are desiccation- and freeze-tolerant and the adults are wingless."

L31. Here and elsewhere the authors say that their subject, P. steinenii, could be a good species for comparative analysis with B. antactica, however that would depend on how close a relative it is. From their phylogeny in Figure 1C is appears that they are very distantly related to each other so presumably these are two independent examples of adaptation to a cold environment. In this case it would be hard to come to much of a conclusion as their routes to cold-resistance might be completely different. This affects the final sentence of the Conclusions too.
> We have rewritten the sentence as follows:
"We present an annotated draft genome of the Antarctic midge, P. steinenii. The genomes of P. steinenii and B. antarctica will aid in the elucidation of evolution under harsh environments and provide new resources for functional genomic analyses of the order Diptera."

L49. The authors use the singular sense to describe the "Specimen of Parachlus steinenii was collected", implying that the entire genome sequence was obtained from a single specimen, however they then describe at least three libraries constructed for the project and it is hard to imagine doing that from a single midge. I presume they mean to say "Specimens …. were collected". Even so it would be good to specify how many individuals were used for each of the three libraries, especially the fragment or paired-end library, because that determines how many different haplotypes might be represented in the assembly. Presumably the jumping or mate-pair libraries were from multiple specimens.
> We used the genomic DNA extracted from twenty adults for genomic libraries and have added the sentence in the manuscript:
"Twenty adults were used for genome sequencing, regardless of gender."

L53. As written this does not make sense as there were apparently two jumping or mate-pair libraries with inserts originally 3 and 5kb long, so it should be plural.
> We have changed "while the other was a jumping library" to "whereas others were jumping libraries."

L54. Again, the authors say "Paired-end libraries were sequenced…", however they describe only a single paired-end library as being constructed.
> We have corrected "libraries" with "library" on line 54.

L60. While technically "expressed sequencing tags", this term is generally not used for modern RNAseq libraries sequenced on ILLUMINA machines, instead these are generally entire transcriptomes. The term ESTs went out with Sanger sequencing.
> We have changed "expressed sequencing tags" to "RNA evidence" on lines 62 and have changed "expressed sequencing tags" to "transcriptome assembly" on line 165.

In Table 1, the authors list three PE300 libraries for RNAseq, however in the text they only mention a "whole body" extraction, so were all three libraries from the same whole body extraction? If so, why three libraries?
> We have added a legend to Table 1 as follows:
"Tree-type libraries were constructed in this study. A PE400 library was constructed as a fragment library for ALLPATHS-LG. Mate-pair libraries (MP3K and MP5K) were also constructed for ALLPATHS-LG assembly. Three PE300 libraries (PE300A, PE300B, and PE300C) were constructed from RNA for gene annotation."
We have changed the manuscript as follows:
"total RNA was extracted from the whole body of 10 adults in three different groups using the RNeasy mini kit (Qiagen, Valencia, CA, USA), according to the manufacturer's instructions."

The English is the description of the Genome Assembly, lines 74-83 is again poor with singular and plural mixed up repeatedly. And what does it mean that "In this assembly, 93.8% of the fragment library was full."?
> We have corrected the singular and plural mix up and we changed "93.8% of the fragment library was full" to "93.8% of the paired-end reads from the fragment library overlapped and merged into one longer read."
Thus, we have rewritten the section as follows:

“Assembly was performed using ALLPATHS-LG for both the fragment library (400 bp) and the jumping libraries (3 kbp and 5 kbp) [4]. This was performed on a 96-processor workstation with an Intel Xeon X7460 2.66 GHz processor, 1 terabyte RAM, and default parameters. For better assembly in ALLPATHS-LG, a larger k-mer size was used with one longer read generated from the paired-end library [4]. As a result, the paired-end reads from the fragment library were designed to overlap, and the insert size of the paired-end library was slightly less than twice the read size [4]. In this assembly, 93.8% of the paired-end reads from the fragment library overlapped and merged into one longer read. The resulting assembly had a total size of 138 Mb, comprising 9,513 contigs, with an N50 contig size of 34,110 bp, and an N50 scaffold size of 168 kb (Table 2). The GC content was 32.2% and the assembly revealed contig coverage of approximately 89 × total read length from the fragment library.”

At this point I would suggest a slight reorganization of the manuscript, placing the Repeat Analysis and Non-coding RNA section before the Gene Annotation sections, which makes more sense as the repeats were then masked for the gene annotation.
> As the reviewer suggested, we have placed the repeat analysis and non-coding RNA section before the gene annotation section.

L151-154 are redundant.
> We have removed the sentence “coding sequences (CDS) … … In this study,”

There is something unsettling about the gene family expansion analysis reported in L194-208 and Table 7. Perhaps it is just that the families identified, such as ID PS0074 for "serine protease" are just one particular family of proteases, but it certainly seems very unlikely, for example, that P steinenii would have no serine proteases. A little more elaboration of these results would be useful.
> We have added more description to the revised manuscript as follows:
“To estimate the average gene expansion/contraction rate and to identify gene families that have undergone significant size changes through evolution [23, 24], we estimated differences in the size of 15,633 orthologs using the program CAFE3.0 [25]. A Newick description of a rooted and bifurcating phylogenetic tree was needed for this analysis. Therefore, we performed phylogenetic analyses among six insects with the protein-coding gene in the orthologous groups. We selected 4,814 orthologous gene sets from the orthologous groups from OrthoMCL using the reciprocal best BLASTP hit criteria. Protein-coding gene sequences were aligned using PRANK (Ver. 130820) under a codon model with the “-DNA and –codon” option [26], poor alignment sites were eliminated using Gblock (Ver. 0.91) under a codon model with the “-t = c” option [27], and the remaining alignment regions were concatenated to be used in the phylogenetic analyses. The phylogenetic tree was constructed using the neighbor-joining method [28] in the MEGA version 6 program [29]. With the resulting phylogenetic tree, we prepared the ultrametric tree of the species, including branch lengths in units of time through TimeTree [30], for the analysis (Figure 1C). We ran the program using $p < 0.05$, and estimated birth (λ) and death (μ) rates were calculated using the program LambdaMu with the “–s” option. We calculated the number of gene gains and losses on each branch of the tree with the “–t” option. Average expansion size of the two Antarctic midges were lower than that of other insects (Figure 1C), and average expansion size of D. melanogaster exhibited the highest score among the six insects. Using $p < 0.0001$ for the family-wide significance value, we expected approximately one significant result by chance and calculated the exact p-values for transitions over every branch. We called individual branches significant at $p < 0.005$ [31]. We identified three and two gene families that were significantly expanded in P. steinenii and B. antarctica, respectively (Table S3).”

In addition, we have changed “serine protease” to “serine protease gd-like.”
