# Supplementary material for: Genome sequencing of the winged midge, Parochlus steinenii, from the Antarctic Peninsula
Source: Gigascience. 2017 Feb 24;6(3):1–8. doi: 10.1093/gigascience/giw009 (PMC5467013; doi:10.1093/gigascience/giw009)
Supplement: Reviewer_1_Report_(Original_Submission).pdf [file giw009_Reviewer_1_Report_(Original_Submission).pdf]

**Reviewer Report**

**Title:** "Genome sequencing of the winged midge, Parochlus steinenii, from the Antarctic Peninsula"

**Version:** Original Submission **Date:** 9/2/2016

**Reviewer name:** Monica Poelchau

**Reviewer Comments to Author:**

This paper describes the genome assembly and annotation of the winged midge, Parochlus steinenii. This species is of particular interest, as it co-occurs in Antarctica with another midge species, Belgica antarctica, but is cold-tolerant; comparative analyses of these two genomes may yield insights into the origins of freeze-tolerance in Belgica antarctica. The data generated and the analyses performed are useful, and should be valuable to the insect comparative genomics community. However, there are a number of uncertainties with the manuscript. Specifically, many details of the analyses are left out, which will make it difficult for others to 1) understand and 2) replicate the analyses performed. It is possible that these details are contained within the 'Supporting data' in the GigaDB database, but I do not have access to these records, and there are no mentions of the Supporting data within the manuscript, except under 'Availability of supporting data'.

Specific Comments:

Abstract - Background

l. 28: "with a compact genome as a result of adaptation to an extreme environment": As far as I know, there are no studies yet that have determined that B. antarctica's small genome size is a result of the extreme environment this insect lives in (the 2014 genome paper doesn't go that far). Please soften the language, or provide a citation that demonstrates a causal relationshiop.

l. 31: change "are cold, but not freeze, tolerant" to "are cold- but not freeze-tolerant,"

Abstract - Conclusions

l. 44: Please change "cold, but not freeze, tolerant" to "cold- but not freeze- tolerant"

Data description - Sequencing

l. 49. How many individuals? Did you determine the sex, or was it a mixed collection? If there were too many to count, did you weigh them? What life history stage?

l. 61: What life history stage? How many insects?

l. 61: Which Qiagen kit was used?

l. 67: Which Fastx program was used?

l. 69: I don't understand what you mean by "data from paired-end trimmed reads with 14 gigabase pairs (Gbp) were obtained". I couldn't find a table legend that explained this, either.

Table 1:

- I couldn't find a table legend.

- the column 'Read lengths' doesn't make sense to me - is this the combined length of all reads?

Data description - Genome assembly

l. 78: "the fragment library should be designed to overlap": Do you mean that the reads from paired-end library overlapped, and were thus combined to generate one longer read?

l. 79: I don't understand what this means: "In this assembly, 93.8% of the fragment library was full".

l. 81: "The resulting assembly had a total size of 137 Mb" - In table 2, you list 130.6 Mb for the contigs and 138 Mb for the scaffolds. Where do you get the number 137 Mb from?

l. 83: How did you calculate the coverage?

Data description - Gene annotation

l. 93: "For proper gene annotation, RNA and protein evidence alignment were used".

- Which RNAs were used? At what step in the Maker program?

- You list the proteins that were used to train Maker in the next paragraph (l. 99-104). Please list them here, instead.

l. 94: What ESTs were used?

l. 96: What transcriptome assembly? Does this line describe the assembly of the RNA data that were generated? Were reads trimmed prior to the assembly? This needs more detail.

l. 111: "This was annotated with the BLASTp results and InterproScan [9]." I'm confused by this sentence - what is "This"? which BLASTp results - is this output from the Blast2Go program, or another analysis? Also, are the InterproScan results part of the Blast2Go analysis?

Data description - Gene annotation for B. antarctica

l. 125 - are the six other species used for protein evidence the same that are listed on l. 99-104?

l. 143: How did the methods for repeat analysis differ between this paper and the B. antarctica genome paper (cited in [5])?

Table 4 - This could probably be moved to a supplement, or condensed.

Data description - Ortholog analysis

Table 5 - This should be moved to a supplement, or condensed. Also, is there supporting data that lists what genes are in which group? It is interesting that D. melanogaster has so many unique proteins, compared to the other 5 species. Can you speculate why?

Data description - Gene structure of Orthologous groups

l. 174 - Are you using the genome size for B. antarctica that was calculated by flow cytometry for this comparison? Perhaps you should use the range calculated from genome sequencing, instead, since this is how you are estimating the P. steinenii genome size.

Data description - GO enrichment test

l. 180: "statistically represented" - do you mean over-represented?

l. 180: "437 orthologous groups" - please change to "437 orthologous groups that are unique to P. steinenii"

l. 181: AgriGO has several analysis tools. Which one did you use, with what parameters?

l. 182: "significant levels of $p = 0.05$" - do you mean "significance levels of $p < 0.05$"?

l. 185: Can you 1) explain what an unfolded protein response is, and how this may be biologically interesting for P. steinenii, and 2) elaborate on why you think an enrichment for genes associated with 'unfolded protein response under stress conditions' in the orthomcl groups that are unique to P. steinenii implies that they evolved independently? Finally, why did you only single out these categories, when many more were enriched for genes in orthomcl groups unique to P. steinenii?

Data description - Likelihood analysis of gene gain and loss

l. 198 - The URL doesn't work.

l. 199 - How did you generate the tree? From what datasets?

l. 218 - Are you going to deposit the genome assembly in a public repository?

General

- I couldn't find any Table legends. These are essential, as the tables by themselves are not descriptive enough.

- It is not clear what supporting data are in the GigaDB database - is there a way to make this obvious to the reader in the manuscript?

**Level of Interest**

Please indicate how interesting you found the manuscript: An article whose findings are important to those with closely related research interests

**Quality of Written English**

Please indicate the quality of language in the manuscript: Not suitable for publication unless extensively edited

**Declaration of Competing Interests**

Please complete a declaration of competing interests, considering the following questions:

- Have you in the past five years received reimbursements, fees, funding, or salary from an organisation that may in any way gain or lose financially from the publication of this manuscript, either now or in the future?
- Do you hold any stocks or shares in an organisation that may in any way gain or lose financially from the publication of this manuscript, either now or in the future?
- Do you hold or are you currently applying for any patents relating to the content of the manuscript?
- Have you received reimbursements, fees, funding, or salary from an organization that holds or has applied for patents relating to the content of the manuscript?
- Do you have any other financial competing interests?
- Do you have any non-financial competing interests in relation to this paper?

If you can answer no to all of the above, write 'I declare that I have no competing interests' below. If your reply is yes to any, please give details below.

I declare that I have no competing interests

I agree to the open peer review policy of the journal. I understand that my name will be included on my report to the authors and, if the manuscript is accepted for publication, my named report including any attachments I upload will be posted on the website along with the authors' responses. I agree for my report to be made available under an Open Access Creative Commons CC-BY license (http://creativecommons.org/licenses/by/4.0/). I understand that any comments which I do not wish to be included in my named report can be included as confidential comments to the editors, which will not be published.

I agree to the open peer review policy of the journal
