# Supplementary material for: Genome sequencing of the winged midge, Parochlus steinenii, from the Antarctic Peninsula
Source: Gigascience. 2017 Feb 24;6(3):1–8. doi: 10.1093/gigascience/giw009 (PMC5467013; doi:10.1093/gigascience/giw009)
Supplement: Reviewer_2_Report_(Original_Submission).pdf [file giw009_Reviewer_2_Report_(Original_Submission).pdf]

Reviewer Report

Title: "Genome sequencing of the winged midge, Parochlus steinenii, from the Antarctic Peninsula"

Version: Original Submission **Date:** 9/5/2016

Reviewer name: Evgeny Zdobnov

Reviewer Comments to Author:

A few comments:

- Table 2: Even though scaffolding greatly improved the assembly there is still a great number of scaffolds (>4,000) and a relatively low scaffold N50, compared to the genome size of this midge (~138 Mbp). In addition, I would say that this unexpected given the amount of sequencing data generated for this insect, which resulted in >100x average contig coverage. I would suggest that the authors comment on it and mention some probable causes for this (e.g. increased repeat content, increased heterozygosity, no mate-pair libraries with an insert of >5 Kbp?).

- Lines 73-83: While the authors mention all the tools and parameters used for genome assembly, there is no mention about the tool they used for scaffolding. I think it would be nice to add this important information, especially since scaffolding contributes to a significant improvement of the assembly.

- Line 92: Why did you only use SNAP for gene prediction? Augustus is known to perform better and can be run from the MAKER pipeline.

- The authors haven't performed an evaluation of their genome assembly or their predicted gene set. Such evaluations are usually done by tools such as BUSCO [Simao et al. 2015], that search for conserved genes in the assembly/gene set.

- Table 5 is unnecessarily long and complicated. First of all, I think that not all the different combinations are necessary to show. I would only include the largest groups and also the most biologically important (certainly no more than 10 groups). I would also suggest that the authors find descriptive names of each group, such as "P. steinenii-specific", or Antarctic midge-specific, or mosquito-specific. I find group names such as "ABC", "ABCDF", "BCEF" to not be human-readable. Last, instead of showing numbers of orthologous groups it would be more meaningful to show number of genes (and maybe show how many of them are transcribed).

Some more, minor comments:

- Line 33: The sentence "In this study..." is isolated does not say much. I would suggest to either delete it, or develop it to something more informative.

The ms focused on "reproducibility of analyses",

while more on the biology of this midge would make the story more exciting.

A few comments:

- Table 1: the first two lines refer to libraries "PE400trim" and "PE400". It's not clear to me if these two libraries are different or the same (with PE400tim simply being the trimmed PE400 library). The authors should clarify this.

- Table 1: Is the column named "Read lengths" showing total read lengths? If so, please rename it.

- Line 180: The authors refer to 437 orthologous groups but it is not clear what these are. Are they the P. steinenii-specific groups? If so, it should be clearly mentioned in the sentence to avoid confusion.

- Table 6: I'm not sure what the columns "number of target genes in term" and "number of genes in terms" mean. Is the former representing the number of genes with a GO term in the species-specific orthologous groups, while the latter represents the same number in the whole gene set? You should make the descriptions more clear.

- Some typos:

- line 90: "...using MAKER2..." --> "using the MAKER2..."

- lines 119-124: Please rephrase the sentence "For RNA evidence...". The first part of the sentence (up to "pipe lines") does not make sense.

- line 168: "...of orthologous gene..." --> "of orthologous genes"

- line 184: Is there something missing in "...and 18 GO terms were identified..."?

- Table 6: in the title of the table delete "were".

- line 195: The sentence "The size..." is not informative at all. I suggest you merge it with the next one.

- lines 199-200: "We performed the program" --> "We ran the program".

- line 205: delete "there"

Level of Interest

Please indicate how interesting you found the manuscript: An article whose findings are important to those with closely related research interests

Quality of Written English

Please indicate the quality of language in the manuscript: Acceptable

Declaration of Competing Interests

Please complete a declaration of competing interests, considering the following questions:

- Have you in the past five years received reimbursements, fees, funding, or salary from an organisation that may in any way gain or lose financially from the publication of this manuscript, either now or in the future?
- Do you hold any stocks or shares in an organisation that may in any way gain or lose financially from the publication of this manuscript, either now or in the future?
- Do you hold or are you currently applying for any patents relating to the content of the manuscript?
- Have you received reimbursements, fees, funding, or salary from an organization that holds or has applied for patents relating to the content of the manuscript?
- Do you have any other financial competing interests?
- Do you have any non-financial competing interests in relation to this paper?

If you can answer no to all of the above, write 'I declare that I have no competing interests' below. If your reply is yes to any, please give details below.

No

I agree to the open peer review policy of the journal. I understand that my name will be included on my report to the authors and, if the manuscript is accepted for publication, my named report including any attachments I upload will be posted on the website along with the authors' responses. I agree for my report to be made available under an Open Access Creative Commons CC-BY license (http://creativecommons.org/licenses/by/4.0/). I understand that any comments which I do not wish to be included in my named report can be included as confidential comments to the editors, which will not be published.

I agree to the open peer review policy of the journal
