# Supplementary material for: Genome sequencing of the winged midge, Parochlus steinenii, from the Antarctic Peninsula
Source: Gigascience. 2017 Feb 24;6(3):1–8. doi: 10.1093/gigascience/giw009 (PMC5467013; doi:10.1093/gigascience/giw009)
Supplement: Reviewer_3_Report_(Original_Submission).pdf [file giw009_Reviewer_3_Report_(Original_Submission).pdf]

Reviewer Report

Title: Genome sequencing of the winged midge, Parochlus steinenii, from the Antarctic Peninsula

Version: Original Submission **Date:** 9/12/2016

Reviewer name: Joanna Kelley

Reviewer Comments to Author:

The article describes the genome assembly of the winged midge Parochlus steineii. There are comparisons to other insect genome assemblies. The genome assembly will provide a useful resource for comparative genome studies. My comments and concerns are listed below.

There are statements in the manuscript that are factually incorrect and must be remedied. The statement that B. antarctica "adults lose their wings" (line 29) is an inaccurate description of the adult wing status. The adults never have wings. Also, the genome assembly of B. antarctica proposes that the small genome is likely due to adaptation to cold environment, however, given that there is no comparative data or study of adaptation and genome size, there is not conclusive evidence that the small genome is itself adaptive.

The concluding sentences of the manuscript state that the mechanisms of freeze tolerance are unknown in B. antarctica. The mechanisms are known and have been explored extensively (see 1979 paper http://onlinelibrary.wiley.com/doi/10.1111/j.1365-3032.1979.tb00171.x/abstract and http://www.ncbi.nlm.nih.gov/pubmed/16424090).

How many individuals were used in the genome assembly?

What is the k-mer analysis estimate of genome size and heterozygosity?

Table 2: Later in the manuscript there is a re-analysis of the B. antarctica genome for direct comparison - that data should be presented in Table 2 instead of quoting from the previous paper.

An additional estimate of genome size - flow cytometry, k-mer analysis, etc - would allow the actual comparison of absolute genome size between the species. The sum of scaffolds in the B. antarctica paper was ~10Mb smaller than the flow cytometry estimated size.

The methods for the ortology analysis are missing (lines 125-127).

The authors should run BUSCO to present results on the putative completeness of the genome assembly.

Was the repeat content re-estimated for the B. antarctica assembly?

GO enrichment test (section starting on line 179) is missing crucial information that the 437 orthologous groups are those genes that are unique to P. steinenii. Please note that they are not orthologs if they are unique to P. steinenii. In this analysis, how many genes had no term? What is the correction for genes belonging to multiple GO levels in the GO enrichment analysis?

What dataset was used for Timetree?

What is the estimated divergence time of the two species?

Minor comments:

Table 1: why are both the pre-and post-trimmed datasets shown for the PE400 data but not the other datasets? Why are three lines shown for the RNAseq PE300 data? Are the three lines from the same library? If so, the amounts can be summed and presented on one line, if not, the differences need to be explained in the table and in the text.

Table 2 and the text are not consistent with regards to the reported size of the genome.

What does "93.8% of the fragment library was full" mean? It is not clear in the text.

Several of the tables should be included as supplementary tables and not in the main text (Table 4 and Table 5, for example).

Table 7 is unclear, what are the 1*, 2* etc?

The use of "expressed sequence tags" to describe RNAseq is incorrect.

The sentence on lines 68-69 does not make sense.

Blast2Go is Blast2GO. Similarly, maker should be capitalized.

Level of Interest

Please indicate how interesting you found the manuscript: An article whose findings are important to those with closely related research interests

Quality of Written English

Please indicate the quality of language in the manuscript: Needs some language corrections before being published

Declaration of Competing Interests

Please complete a declaration of competing interests, considering the following questions:

- Have you in the past five years received reimbursements, fees, funding, or salary from an organisation that may in any way gain or lose financially from the publication of this manuscript, either now or in the future?
- Do you hold any stocks or shares in an organisation that may in any way gain or lose financially from the publication of this manuscript, either now or in the future?
- Do you hold or are you currently applying for any patents relating to the content of the manuscript?
- Have you received reimbursements, fees, funding, or salary from an organization that holds or has applied for patents relating to the content of the manuscript?
- Do you have any other financial competing interests?
- Do you have any non-financial competing interests in relation to this paper?

If you can answer no to all of the above, write 'I declare that I have no competing interests' below. If your reply is yes to any, please give details below.

I declare that I have no competing interests

I agree to the open peer review policy of the journal. I understand that my name will be included on my report to the authors and, if the manuscript is accepted for publication, my named report including any attachments I upload will be posted on the website along with the authors' responses. I agree for my report to be made available under an Open Access Creative Commons CC-BY license (http://creativecommons.org/licenses/by/4.0/). I understand that any comments which I do not wish to be included in my named report can be included as confidential comments to the editors, which will not be published.

I agree to the open peer review policy of the journal
