# Supplementary material for: Genome sequencing of the winged midge, Parochlus steinenii, from the Antarctic Peninsula
Source: Gigascience. 2017 Feb 24;6(3):1–8. doi: 10.1093/gigascience/giw009 (PMC5467013; doi:10.1093/gigascience/giw009)
Supplement: Reviewer_4_Report_(Original_Submission).pdf [file giw009_Reviewer_4_Report_(Original_Submission).pdf]

Reviewer Report

Title: "Genome sequencing of the winged midge, Parochlus steinenii, from the Antarctic Peninsula"

Version: Original Submission **Date:** 9/12/2016

Reviewer name: Hugh Robertson

Reviewer Comments to Author:

The authors describe their sequencing of this midge genome and a pretty typical set of metrics of evaluating it, but not much more. I understand this is acceptable for a Data Note, so instead have focused this review on making the work more readable and interpretable.

L28. In the Background of the Abstract, the authors says that "B. antartica has unusual characteristics with a compact genome as a result of adaptation to an extreme environment". I don't think there is any evidence that the compactness of that genome has anything to do with the extreme Antarctic environment, it could just be a coincidence. Perhaps other members of that genus or that lineage of midges has similarly tiny genomes, and even if they don't, one would require study of many independent origins of cold-hardiness to say small genomes result from adaptation to extreme environments.

L31. Here and elsewhere the authors say that their subject, P. steinenii, could be a good species for comparative analysis with B. antactica, however that would depend on how close a relative it is. From their phylogeny in Figure 1C is appears that they are very distantly related to each other so presumably these are two independent examples of adaptation to a cold environment. In this case it would be hard to come to much of a conclusion as their routes to cold-resistance might be completely different. This affects the final sentence of the Conclusions too.

L49. The authors use the singular sense to describe the "Specimen of Parachlus steinenii was collected", implying that the entire genome sequence was obtained from a single specimen, however they then describe at least three libraries constructed for the project and it is hard to imagine doing that from a single midge. I presume they mean to say "Specimens …. were collected". Even so it would be good to specify how many individuals were used for each of the three libraries, especially the fragment or paired-end library, because that determines how many different haplotypes might be represented in the assembly. Presumably the jumping or mate-pair libraries were from multiple specimens.

L53. As written this does not make sense as there were apparently two jumping or mate-pair libraries with inserts originally 3 and 5kb long, so it should be plural.

L54. Again, the authors say "Paired-end libraries were sequenced…", however they describe only a single paired-end library as being constructed.

L60. While technically "expressed sequencing tags", this term is generally not used for modern RNAseq libraries sequenced on ILLUMINA machines, instead these are generally entire transcriptomes. The term ESTs went out with Sanger sequencing.

In Table 1, the authors list three PE300 libraries for RNAseq, however in the text they only mention a "whole body" extraction, so were all three libraries from the same whole body extraction? If so, why three libraries?

The English is the description of the Genome Assembly, lines 74-83 is again poor with singular and plural mixed up repeatedly. And what does it mean that "In this assembly, 93.8% of the fragment library was full."?

At this point I would suggest a slight reorganization of the manuscript, placing the Repeat Analysis and Non-coding RNA section before the Gene Annotation sections, which makes more sense as the repeats were then masked for the gene annotation.

L151-154 are redundant.

There is something unsettling about the gene family expansion analysis reported in L194-208 and Table 7. Perhaps it is just that the families identified, such as ID PS0074 for "serine protease" are just one particular family of proteases, but it certainly seems very unlikely, for example, that P steinenii would have no serine proteases. A little more elaboration of these results would be useful.

Level of Interest

Please indicate how interesting you found the manuscript: An article whose findings are important to those with closely related research interests

Quality of Written English

Please indicate the quality of language in the manuscript: Needs some language corrections before being published

Declaration of Competing Interests

Please complete a declaration of competing interests, considering the following questions:

- Have you in the past five years received reimbursements, fees, funding, or salary from an organisation that may in any way gain or lose financially from the publication of this manuscript, either now or in the future?
- Do you hold any stocks or shares in an organisation that may in any way gain or lose financially from the publication of this manuscript, either now or in the future?
- Do you hold or are you currently applying for any patents relating to the content of the manuscript?
- Have you received reimbursements, fees, funding, or salary from an organization that holds or has applied for patents relating to the content of the manuscript?
- Do you have any other financial competing interests?
- Do you have any non-financial competing interests in relation to this paper?

If you can answer no to all of the above, write 'I declare that I have no competing interests' below. If your reply is yes to any, please give details below.

I declare that I have no competing interests.

I agree to the open peer review policy of the journal. I understand that my name will be included on my report to the authors and, if the manuscript is accepted for publication, my named report including any attachments I upload will be posted on the website along with the authors' responses. I agree for my report to be made available under an Open Access Creative Commons CC-BY license (http://creativecommons.org/licenses/by/4.0/). I understand that any comments which I do not wish to be included in my named report can be included as confidential comments to the editors, which will not be published.

I agree to the open peer review policy of the journal
